# Supplementary material for: Memory CD4 T cell subsets are kinetically heterogeneous and replenished from naive T cells at high levels
Source: eLife. 2017 Mar 10;6:e23013. doi: 10.7554/eLife.23013 (PMC5426903; doi:10.7554/eLife.23013)
Supplement: Figure 2—source data 2. — DOI: http://dx.doi.org/10.7554/eLife.23013.008 [file elife-23013-fig2-data2.zip › SourceAnalysisELife.html]

SourceAnalysisELife


We estimate the rate at which CD4+ T cells transition from naive to central and effector memory compartments, and an alternative model where T cells transition from central to effector memory, all in the absence of deliberate infection. First we fit some simple curves to the counts of the raw number of cells and the degree of chimerism observed in the source compartments, Naive or Central Memory depending on the model. These fitted curves are used as givens in a mechanistic ODE model of memory population dynamics. Using this mechanistic model we estimate parameters of interest: The raw flow from source to memory populations, the rate of decay of this inflow, and the proportional size of this inflow measured as a total fraction of the memory compartment which is comprised of novel inflow at a given time.

In [1]:

```
# these are the tools we'll be using
#standard numerical manipulation package
import numpy as np
print('numpy version: ' + str(np.__version__))

# data frame package
import pandas as pd
print('pandas version: ' + str(pd.__version__))

# non-linear least squares optimizer
import scipy
print('scipy version: ' + str(scipy.__version__))
from scipy.optimize import least_squares
# numerical integrator
from scipy.integrate import odeint

#plotting tools
%matplotlib inline
import matplotlib
print('matplotlib version: ' + str(matplotlib.__version__))
import matplotlib.pyplot as plt
import seaborn as sns
print('seaborn version: ' + str(sns.__version__))
from scipy.stats import gaussian_kde

# tools to take advantage of parallel processing, 
from joblib import Parallel, delayed
import multiprocessing

# debugging tools
import time as time_module
import pdb
```

```
numpy version: 1.11.3
pandas version: 0.19.2
scipy version: 0.18.1
matplotlib version: 2.0.0
seaborn version: 0.7.1
```

In [2]:

```
#We use these functions to transform data and parameters
def logit(p):
    return np.log(p/(1-p))

def sigmoid(q):
    return (np.exp(q) / (1 + np.exp(q)))
```

We are going to use different subsets of data to achieve different things.

- Fit exponential curve of naive counts
- Fit exponential curve of central memory counts
- Fit logisitic curve of naive chimerism
- Fit fit logistic curve of central memory chimerism
- Use these fitted curves of potential source populations to fit a mechanistic ode model of chimerism and counts in central and effector memory compartment
  Each of these task may require a slightly different subset of the data.

In [3]:

```
# The data for exponential model of Naive counts.
# Lots of data here as all of the control mice can be included
ddNaiCount = pd.read_csv('dataForNaiCounts.csv')
```

In [4]:

```
# Data for exponential model of Central Memory Counts.
# Fewer data points here as not all controls included a stain 
# for CD62L to differentiate effector from central memory
ddCmSplineCount = pd.read_csv('dataForCmCurveCounts.csv')
```

In [5]:

```
# Data for logistic model of Naive Chimerism
# All chimeras past the age of DP1 saturation, and where saturated
# chimerism is greater than 0.1 are used here.
ddNaiChi = pd.read_csv('dataForNaiChi.csv')
```

In [6]:

```
# Data for the logistic model of Central memory chimerism
# All the mice used for the Naive Chimerism, that also have the CD62L
# stain
ddCmSplineChi = pd.read_csv('dataForCmCurveChi.csv')
```

In [7]:

```
# The data for the target time courses
# Same mice as used for Central Memory Chimerism
ddTarget = pd.read_csv('dataForEmCmTargets.csv')
```

In [8]:

```
ddTarget
```

Out[8]:

|  | mouse.age.days | age.at.bmt | days.post.bmt | TOTAL.4Tem | TOTAL.4Tcm | log.TOTAL.4Tem | log.TOTAL.4Tcm | NCHI.4Tem | NCHI.4Tcm | logit.NCHI.4Tem | logit.NCHI.4Tcm |
| --- | --- | --- | --- | --- | --- | --- | --- | --- | --- | --- | --- |
| 0 | 98 | 56 | 42 | 6111900.0 | 1451300.0 | 15.625748 | 14.187970 | 0.034921 | 0.102575 | -3.319110 | -2.168935 |
| 1 | 98 | 56 | 42 | 7018500.0 | 1539800.0 | 15.764060 | 14.247163 | 0.012106 | 0.044143 | -4.401894 | -3.075173 |
| 2 | 98 | 56 | 42 | 5800400.0 | 929400.0 | 15.573437 | 13.742294 | 0.024796 | 0.054565 | -3.671954 | -2.852261 |
| 3 | 98 | 56 | 42 | 3671000.0 | 825300.0 | 15.115975 | 13.623502 | 0.038990 | 0.088427 | -3.204691 | -2.332992 |
| 4 | 119 | 56 | 63 | 4482700.0 | 768200.0 | 15.315736 | 13.551805 | 0.067922 | 0.136647 | -2.619057 | -1.843419 |
| 5 | 119 | 56 | 63 | 3433800.0 | 678700.0 | 15.049178 | 13.427934 | 0.128496 | 0.196892 | -1.914324 | -1.405831 |
| 6 | 119 | 56 | 63 | 6698000.0 | 816900.0 | 15.717320 | 13.613272 | 0.113213 | 0.272551 | -2.058334 | -0.981718 |
| 7 | 140 | 56 | 84 | 4712000.0 | 490600.0 | 15.365623 | 13.103384 | 0.124862 | 0.246284 | -1.947169 | -1.118533 |
| 8 | 140 | 56 | 84 | 8667000.0 | 899000.0 | 15.975033 | 13.709038 | 0.134108 | 0.246192 | -1.865117 | -1.119027 |
| 9 | 140 | 56 | 84 | 8303000.0 | 628300.0 | 15.932127 | 13.350773 | 0.166391 | 0.308763 | -1.611423 | -0.805907 |
| 10 | 140 | 56 | 84 | 8082000.0 | 733100.0 | 15.905150 | 13.505037 | 0.112203 | 0.212389 | -2.068433 | -1.310583 |
| 11 | 168 | 56 | 112 | 10935000.0 | 1127200.0 | 16.207479 | 13.935247 | 0.178582 | 0.319898 | -1.525984 | -0.754239 |
| 12 | 168 | 56 | 112 | 12016000.0 | 877800.0 | 16.301750 | 13.685174 | 0.221583 | 0.408299 | -1.256468 | -0.371000 |
| 13 | 168 | 56 | 112 | 14375000.0 | 1031100.0 | 16.481001 | 13.846137 | 0.218973 | 0.404298 | -1.271659 | -0.387590 |
| 14 | 168 | 56 | 112 | 10137000.0 | 608500.0 | 16.131703 | 13.318752 | 0.212954 | 0.436674 | -1.307208 | -0.254671 |
| 15 | 196 | 56 | 140 | 11478000.0 | 1415600.0 | 16.255943 | 14.163064 | 0.187544 | 0.334781 | -1.466046 | -0.686642 |
| 16 | 196 | 56 | 140 | 18936000.0 | 1930400.0 | 16.756575 | 14.473238 | 0.249316 | 0.440460 | -1.102262 | -0.239297 |
| 17 | 259 | 56 | 203 | 8687000.0 | 476400.0 | 15.977338 | 13.074013 | 0.286419 | 0.359729 | -0.912838 | -0.576540 |
| 18 | 259 | 56 | 203 | 6419000.0 | 291400.0 | 15.674773 | 12.582452 | 0.406939 | 0.533788 | -0.376633 | 0.135357 |
| 19 | 259 | 56 | 203 | 7779000.0 | 317400.0 | 15.866938 | 12.667918 | 0.479330 | 0.628520 | -0.082725 | 0.525873 |
| 20 | 259 | 56 | 203 | 8746000.0 | 398600.0 | 15.984107 | 12.895714 | 0.274034 | 0.396859 | -0.974252 | -0.418568 |
| 21 | 420 | 56 | 364 | 9073000.0 | 309200.0 | 16.020814 | 12.641744 | 0.283807 | 0.359750 | -0.925657 | -0.576448 |
| 22 | 420 | 56 | 364 | 18154000.0 | 538000.0 | 16.714401 | 13.195614 | 0.474098 | 0.497273 | -0.103700 | -0.010909 |
| 23 | 420 | 56 | 364 | 10335000.0 | 300800.0 | 16.151047 | 12.614201 | 0.490664 | 0.676968 | -0.037349 | 0.739871 |

We model the naive CD4+ population as exponentially decaying. We use least squares to estimate its parameters. We similarly model number the CD4+ central memory cells, also modeled as simple exponential decay.

In [9]:

```
def exp_model(params, t):
    S_0, R = params
    return S_0*np.exp(-R*t)

def T_log_residuals_func(trans_params, t, log_obs):
    log_S_0, R = trans_params
    S_0 = np.exp(log_S_0)
    params = S_0, R
    model = exp_model(params, t)
    residuals = log_obs - np.log(model)
    return residuals
```

In [10]:

```
def estimate_source_counts_curve(data, source_pop,
                                 params_guess = [np.log(1e+7), -1e-2],
                                 log_residuals=1, 
                                 verbose=False):
    #Source pop will be one of TOTAL.4nai or TOTAL.4Tcm
    T = data[source_pop]
    log_T = data['log.'+source_pop]
    # when log_residuals = 1 this just gives the original data,
    # when it is the actual log residuals we get a bootstraped sample
    np.random.seed() # put this here so that works in parallel
    fake_log_T = log_T + np.random.choice(log_residuals, len(log_T))
    age = data['mouse.age.days']
    # This is analytically tractable, but simplicity we use 
    # the same least squares optimizer throughout
    T_result = least_squares(T_log_residuals_func, params_guess, method='trf', args=(age, fake_log_T))
    trans_params_hat = T_result['x']
    params_hat = np.exp(trans_params_hat[0]), trans_params_hat[1]
    T_residuals = 0
    T_log_residuals = 0
    
    if verbose:
        T_hat = exp_model(params_hat, age)
        T_residuals = T - T_hat
        T_log_residuals = np.log(T) - np.log(T_hat)
        smoothAge = np.linspace(np.min(age), np.max(age),500)
        T_hat_smooth = exp_model(params_hat, smoothAge)
        fig, ax = plt.subplots()
        ax.scatter(age, T)
        ax.plot(smoothAge, T_hat_smooth, label=source_pop)
        ax.set_xlabel('mouse age (days)')
        ax.set_ylabel('cell counts')
        ax.set_title('exponential model of '+ source_pop + ' cell counts over time')
        
    return (params_hat, T_residuals, T_log_residuals)
```

In [11]:

```
(NT_params_hat, 
 NT_residuals, 
 NT_log_residuals) = estimate_source_counts_curve(ddNaiCount,
                                                  'TOTAL.4nai',
                                                  verbose=True)
```

In [12]:

```
NT_params_hat
```

Out[12]:

```
(51944361.008761428, 0.0052518114635097188)
```

In [13]:

```
(CT_spline_params_hat,
 CT_residuals,
 CT_log_residuals) = estimate_source_counts_curve(ddCmSplineCount,
                                                  'TOTAL.4Tcm',
                                                  verbose=True)
```

In [14]:

```
CT_spline_params_hat
```

Out[14]:

```
(1078746.8994628179, 0.002293638987108642)
```

Now we use bootstraps (resampling the residuals) to establish confidence intervals for these count spline parameter estimates.

In [15]:

```
num_bootstraps = 10000
inputs = range(num_bootstraps)
num_cores = multiprocessing.cpu_count()
```

In [16]:

```
def NT_boot_wrap(i):
    (NT_params_boot,
     NT_residuals_boot,
     NT_log_residuals_boot) = estimate_source_counts_curve(ddNaiCount,
                                                           'TOTAL.4nai',
                                                           params_guess = (np.log(NT_params_hat[0]), NT_params_hat[1]),
                                                           log_residuals=NT_log_residuals,
                                                           verbose=False)
    return (NT_params_boot)

results = Parallel(n_jobs=num_cores)(delayed(NT_boot_wrap)(i) for i in inputs)
NT_bootstrap_params = np.array(results).T
```

In [17]:

```
def CT_boot_wrap(i):
    (CT_spline_params_boot, 
     CT_spline_residuals_boot, 
     CT_spline_log_residuals_boot) = estimate_source_counts_curve(ddCmSplineCount,
                                                                  'TOTAL.4Tcm',
                                                                  params_guess = (np.log(CT_spline_params_hat[0]), CT_spline_params_hat[1]),
                                                                  log_residuals= CT_log_residuals,
                                                                  verbose=False)
    return (CT_spline_params_boot)

results = Parallel(n_jobs=num_cores)(delayed(CT_boot_wrap)(i) for i in inputs)
CT_spline_bootstrap_params = np.array(results).T
```

In [18]:

```
def make_count_CI(bootstrap_params, source_pop, verbose=True):
    num_bootstraps = len(bootstrap_params[0,:])
    sorted_straps = np.zeros((2,num_bootstraps))
    sorted_straps[0,:] = np.sort(bootstrap_params[0,:])
    sorted_straps[1,:] = np.sort(bootstrap_params[1,:])
    T_lowerBounds = sorted_straps[:, np.int(np.ceil(0.025 * num_bootstraps))]
    T_upperBounds = sorted_straps[:, np.int(np.floor(0.975 * num_bootstraps))]
    if verbose:
        print('Exponential model of counts of ' + source_pop + ', parameter S_0 has a 95 percent Bootstrap interval of (%.6f, %.6f)' % (T_lowerBounds[0], T_upperBounds[0]))
        print('Parameter r has a 95 percent confidence interval of  (%.6f, %.6f).' % (T_lowerBounds[1], T_upperBounds[1]))
    return T_lowerBounds, T_upperBounds
```

In [19]:

```
NT_lowerBounds, NT_upperBounds = make_count_CI(NT_bootstrap_params, 
                                               'CD4+ Naive')
```

```
Exponential model of counts of CD4+ Naive, parameter S_0 has a 95 percent Bootstrap interval of (43691445.853744, 61964058.996408)
Parameter r has a 95 percent confidence interval of  (0.004548, 0.005971).
```

In [20]:

```
CT_lowerBounds, CT_upperBounds = make_count_CI(CT_spline_bootstrap_params, 
                                               'CD4+ Central Memory')
```

```
Exponential model of counts of CD4+ Central Memory, parameter S_0 has a 95 percent Bootstrap interval of (820164.206650, 1439008.085862)
Parameter r has a 95 percent confidence interval of  (0.000849, 0.003666).
```

Lastly we see how these bootstraps reflect our uncertainty around the fit counts curve in the space of the data.

In [21]:

```
def compute_T_hat_bounds(data, source_pop, params_hat, bootstrap_params, verbose=True):
    T = data[source_pop]
    age = data['mouse.age.days']
    smoothAge = np.arange(np.min(age), np.max(age)+1)
    T_hat_bounds = np.zeros((2,len(smoothAge)))
    num_bootstraps = bootstrap_params.shape[-1]
    bound_indices = [np.int(np.floor(0.975 * num_bootstraps)), 
                     np.int(np.ceil(0.025 * num_bootstraps))]

    for ii, sampleAge in enumerate(smoothAge):
        T_param_samples = bootstrap_params
        T_t = exp_model(T_param_samples, sampleAge)
        sorted_T_t = np.sort(T_t)
        T_hat_bounds[:,ii] = sorted_T_t[bound_indices]
    if verbose:
        # plot the data the fit and the confidence bounds
        T_hat_smooth = exp_model(params_hat, smoothAge)
        fig, ax = plt.subplots()
        ax.fill_between(smoothAge, T_hat_bounds[1,:], T_hat_bounds[0,:], 
                        facecolor=sns.color_palette()[4], interpolate=True)
        ax.scatter(age, T)
        ax.plot(smoothAge, T_hat_smooth, label=source_pop)
        ax.set_xlabel('mouse age (days)')
        ax.set_ylabel('cell counts')
        if source_pop == 'TOTAL.4nai':
            ax.set_title('Exponential Model of CD4+ Naive Population')
            #plt.savefig('Exp4nai.pdf', format='pdf')
        else:
            ax.set_title('Exponential model of CD4+ Central Memory Population')
            #plt.savefig('Exp4Tcm.pdf', format='pdf')
        
        # make nice csv of the numbers used to make the picture 
        CountSplineDF = pd.DataFrame()
        CountSplineDF['age.days'] = smoothAge
        CountSplineDF['source.counts.upp'] = T_hat_bounds[0,:]
        CountSplineDF['source.counts.fit'] = T_hat_smooth
        CountSplineDF['source.counts.low'] = T_hat_bounds[1,:]
        #CountSplineDF.to_csv(source_pop.replace('TOTAL.4','')+'CountSpline.csv')
    return T_hat_bounds
```

In [22]:

```
NT_hat_bounds = compute_T_hat_bounds(ddNaiCount, 'TOTAL.4nai', 
                                     NT_params_hat, NT_bootstrap_params)
```

In [23]:

```
CT_hat_bounds = compute_T_hat_bounds(ddCmSplineCount, 'TOTAL.4Tcm', 
                                     CT_spline_params_hat, CT_spline_bootstrap_params)
```

Now do this all again, but this time for the chimerism.

In [24]:

```
def logistic_trans_to_raw(trans_params):
    log_CHI_0, log_CHI_T, log_r = trans_params
    raw_params = np.exp(log_CHI_0), np.exp(log_CHI_T), np.exp(log_r)
    return raw_params

def logistic_raw_to_trans(raw_params):
    CHI_0, CHI_T, r = raw_params
    trans_params = np.log(CHI_0), np.log(CHI_T), np.log(r)
    return trans_params

def logistic_model(raw_params, t):
    CHI_0, CHI_T, r = raw_params
    return (CHI_T / (1.0 + np.exp(r*-t)*(CHI_T - CHI_0)/CHI_0))

def CHI_logit_residuals_func(trans_params, t, logit_obs):
    raw_params = logistic_trans_to_raw(trans_params)
    model = logistic_model(raw_params, t)
    residuals = logit_obs - logit(model)
    return residuals
```

In [25]:

```
def estimate_source_chimerism_spline(data, source_pop, raw_params_guess = [0.05, 0.6, 0.02], logit_residuals=1, verbose=False):
    #for us source pop will be one of NCHI.4nai of NCHI.4Tcm
    CHI = data[source_pop]
    logit_CHI = data['logit.'+source_pop]
    np.random.seed() # so that we can use it in the bootstraps
    fake_logit_CHI = logit_CHI + np.random.choice(logit_residuals, len(logit_CHI))
    postBMT = data['days.post.bmt']
    trans_params_guess = logistic_raw_to_trans(raw_params_guess)
    CHI_result = least_squares(CHI_logit_residuals_func, trans_params_guess, method='trf', args=(postBMT, fake_logit_CHI))
    trans_params_hat = CHI_result['x']
    raw_params_hat = logistic_trans_to_raw(trans_params_hat)
    CHI_residuals = 0
    CHI_logit_residuals = 0
    
    if verbose:
        CHI_hat = logistic_model(raw_params_hat, postBMT)
        CHI_residuals = CHI - CHI_hat
        CHI_logit_residuals = logit(CHI) - logit(CHI_hat)
        smoothPostBMT = np.linspace(np.min(postBMT), np.max(postBMT),500)
        CHI_hat_smooth = logistic_model(raw_params_hat, smoothPostBMT)
        fig, ax = plt.subplots()
        ax.scatter(postBMT, CHI)
        ax.plot(smoothPostBMT, CHI_hat_smooth, label=source_pop)
        ax.set_xlabel('days poast bone marrow translplant')
        ax.set_ylabel('normalized chimerism')
        ax2 = ax.twiny()
        ax2.set_xlim(ax.get_xlim())
        ax2.set_xticklabels((ax.get_xticks() + 56).astype(int))  #(56 is the age in days at bone marrow transplant)
        ax2.set_xlabel('mouse age in days')
        plt.title('logistic model of '+ source_pop + ' chimerism over time', y=1.1)
        
    return (raw_params_hat, CHI_residuals, CHI_logit_residuals)
```

In [26]:

```
(NCHI_params_hat, 
 NCHI_residuals, 
 NCHI_logit_residuals) = estimate_source_chimerism_spline(ddNaiChi,
                                                          'NCHI.4nai',
                                                          verbose=True)
```

In [27]:

```
NCHI_params_hat #chi_0, chi_T, R
```

Out[27]:

```
(0.091665457418670909, 0.8468213775789708, 0.038351038526788499)
```

In [28]:

```
(CCHI_spline_params_hat, 
 CCHI_residuals, 
 CCHI_logit_residuals) = estimate_source_chimerism_spline(ddCmSplineChi,
                                                          'NCHI.4Tcm',
                                                          verbose=True)
```

In [29]:

```
CCHI_spline_params_hat
```

Out[29]:

```
(0.013056174492123481, 0.47768765096089788, 0.045381221277780028)
```

Now we use bootstraps (resampling the residuals) to establish confidence intervals for these chimerism parameter estimates.

In [30]:

```
num_bootstraps = 10000
inputs = range(num_bootstraps)
num_cores = multiprocessing.cpu_count()
```

In [31]:

```
def NCHI_boot_wrap(i):
    (NCHI_params_boot,
     NCHI_residuals_boot,
     NCHI_log_residuals_boot) = estimate_source_chimerism_spline(ddNaiChi,
                                                                 'NCHI.4nai',
                                                                 raw_params_guess=NCHI_params_hat,
                                                                 logit_residuals=NCHI_logit_residuals,
                                                                 verbose=False)
    return (NCHI_params_boot)


results = Parallel(n_jobs=num_cores)(delayed(NCHI_boot_wrap)(i) for i in inputs)
NCHI_bootstrap_params = np.array(results).T
```

In [32]:

```
def CCHI_boot_wrap(i):
    (CCHI_spline_params_boot, 
     CCHI_spline_residuals_boot, 
     CCHI_spline_log_residuals_boot) = estimate_source_chimerism_spline(ddCmSplineChi,
                                                                        'NCHI.4Tcm',
                                                                        raw_params_guess=CCHI_spline_params_hat,
                                                                        logit_residuals= CCHI_logit_residuals,
                                                                        verbose=False)
    return (CCHI_spline_params_boot)

results = Parallel(n_jobs=num_cores)(delayed(CCHI_boot_wrap)(i) for i in inputs)
CCHI_spline_bootstrap_params = np.array(results).T
```

In [33]:

```
def make_chimerism_CI(bootstrap_params, source_pop, verbose=True):
    num_bootstraps = bootstrap_params.shape[-1]
    sorted_straps = np.zeros((3,num_bootstraps))
    sorted_straps[0,:] = np.sort(bootstrap_params[0,:])
    sorted_straps[1,:] = np.sort(bootstrap_params[1,:])
    sorted_straps[2,:] = np.sort(bootstrap_params[2,:])
    CHI_lowerBounds = sorted_straps[:, np.int(np.ceil(0.025 * num_bootstraps))]
    CHI_upperBounds = sorted_straps[:, np.int(np.floor(0.975 * num_bootstraps))]
    if verbose:
        print('For the logistic model of chimerism of ' + source_pop + ', parameter CHI_0 has a 95 percent confidence interval of (%.3g, %.3g)' % (CHI_lowerBounds[0], CHI_upperBounds[0]))
        print('parameter CHI_T has a 95 percent confidence interval of (%.4g, %.4g).' % (CHI_lowerBounds[1], CHI_upperBounds[1]))
        print('and parameter r has a 95 percent confidence interval of  (%.4g, %.4g).' % (CHI_lowerBounds[2], CHI_upperBounds[2]))
    return CHI_lowerBounds, CHI_upperBounds
```

In [34]:

```
NCHI_lowerBounds, NCHI_upperBounds = make_chimerism_CI(NCHI_bootstrap_params, 'CD4+ Naive')
CCHI_lowerBounds, CCHI_upperBounds = make_chimerism_CI(CCHI_spline_bootstrap_params, 
                                               'CD4+ Central Memory')
```

```
For the logistic model of chimerism of CD4+ Naive, parameter CHI_0 has a 95 percent confidence interval of (0.00977, 0.183)
parameter CHI_T has a 95 percent confidence interval of (0.7944, 0.8843).
and parameter r has a 95 percent confidence interval of  (0.02687, 0.0829).
For the logistic model of chimerism of CD4+ Central Memory, parameter CHI_0 has a 95 percent confidence interval of (0.00333, 0.0342)
parameter CHI_T has a 95 percent confidence interval of (0.4098, 0.5615).
and parameter r has a 95 percent confidence interval of  (0.02953, 0.07247).
```

Lastly we see how these bootstraps reflect our uncertainty around our fit chimerism curve in the space of the data.

In [35]:

```
def compute_CHI_hat_bounds(data, source_pop, params_hat, bootstrap_params, verbose=True):
    CHI = data[source_pop]
    postBMT = data['days.post.bmt'] # days post bone marrow transplant
    smoothPostBMT = np.arange(np.min(postBMT), np.max(postBMT)+1)
    CHI_hat_bounds = np.zeros((2,len(smoothPostBMT)))
    num_bootstraps = bootstrap_params.shape[-1]
    bound_indices = [np.int(np.floor(0.975 * num_bootstraps)), 
                     np.int(np.ceil(0.025 * num_bootstraps))]
    for ii, samplePostBMT in enumerate(smoothPostBMT):
        CHI_param_samples = bootstrap_params
        CHI_t = logistic_model(CHI_param_samples, samplePostBMT)
        sorted_CHI_t = np.sort(CHI_t)
        CHI_hat_bounds[:,ii] = sorted_CHI_t[bound_indices]
    if verbose:
        # plot the data the fit and the confidence bounds
        CHI_hat_smooth = logistic_model(params_hat, smoothPostBMT)
        fig, ax = plt.subplots()
        ax.fill_between(smoothPostBMT, CHI_hat_bounds[1,:], CHI_hat_bounds[0,:], 
                        facecolor=sns.color_palette()[4], interpolate=True)
        ax.scatter(postBMT, CHI)
        ax.plot(smoothPostBMT, CHI_hat_smooth, label=source_pop)
        ax.set_xlabel('days post bone marrow transplant')
        ax.set_ylabel('normalized chimerism')
        ax2 = ax.twiny()
        ax2.set_xlim(ax.get_xlim())
        ax2.set_xticklabels((ax.get_xticks() + 56).astype(int))  #(56 is the age in days at bone marrow transplant)
        ax2.set_xlabel('mouse age in days')
        if source_pop == 'NCHI.4nai':
            plt.title('Logistic model of CD4+ Chimerism', y=1.1)
            #plt.savefig('Logistic4nai.pdf', format='pdf')
        else:
            plt.title('logistic model of '+ source_pop.replace('NCHI.','') + ' chimerism over time', y=1.1)
            #plt.savefig('Logistic4Tcm.pdf', format='pdf')
        # make nice csv of the numbers used to make the picture 
        #ChiSplineDF = pd.DataFrame()
        #ChiSplineDF['age.days'] = smoothPostBMT
        #ChiSplineDF['source.counts.upp'] = CHI_hat_bounds[0,:]
        #ChiSplineDF['source.counts.fit'] = CHI_hat_smooth
        #ChiSplineDF['source.counts.low'] = CHI_hat_bounds[1,:]
        #ChiSplineDF.to_csv(source_pop.replace('NCHI.4','')+'ChiSpline.csv')
    return CHI_hat_bounds
```

In [36]:

```
NCHI_hat_bounds = compute_CHI_hat_bounds(ddNaiChi, 'NCHI.4nai', 
                                         NCHI_params_hat, NCHI_bootstrap_params)
```

In [37]:

```
CCHI_hat_bounds = compute_CHI_hat_bounds(ddCmSplineChi, 'NCHI.4Tcm', 
                                         CCHI_spline_params_hat, CCHI_spline_bootstrap_params)
```

We will use these approximate interpolating curves of source population counts and chimerism as givens when fitting and ODE model of the kinetics of source flow into memory. The dynamics of the Memory host, $M\_{\text{host}}$, and donor, $M\_{\text{donor}}$, populations are modeled as

\begin{align}
\frac{ \text{d} M\_{\text{host}}} {\text{d} t} & = \gamma S\_{\text{host}}(t) -\lambda M\_{\text{host}}(t) \\
\frac{\text{d} M\_{\text{donor}}} {\text{d} t} & = \gamma S\_{\text{donor}}(t) -\lambda M\_{\text{donor}}(t).
\end{align}

The rate $\gamma$ is the product of the per capita rate of egress of cells from the precursor population $S$ and the net effect of any expansion and/or contraction that takes place during the transition into memory.
The rate $\lambda$ is the intrinsic per capita expansion/contraction of the memory population due to division, death and differentiation.

We define the memory chimerism to be the fraction of cells that are donor-derived in the memory population,
$$ \chi\_\text{M} = \frac{M\_\text{donor}}{M\_\text{donor} + M\_\text{host}}. $$
This differs among age-matched animals due to variation in the degree of HSC depletion with busulfan treatment.
We normalise the memory chimerism to that in the thymic precursor population DP1, $\chi\_\text{DP1}$, which is stable by approximately 6 weeks post-BMT.

The equations can then be re-cast in forms that do not depend on the degree of HSC depletion and so are applicable across mice in the experimental cohort:
\begin{align}
\frac{\text{d}M}{\text{d}t} = & \gamma S(t) - \lambda M(t) \
\frac{\text{d}\rho*{\text{M}}}{\text{d}t} = & \frac{\gamma S(t)}{M(t)} \left( \rho*\text{S}(t) - \rho*\text{M}(t) \right)
\end{align}
where $M=M*{\text{host}} + M*{\text{donor}}$, and $\rho*{M} = \chi*\text{M}/\chi*\text{DP1}$ and $\rho*\text{S}= \chi*{S}/\chi\_\text{DP1}$ are the normalised donor chimerism in the memory and source populations respectively.

For the declining recruitment model we allow the per capita source to memory conversion rate to vary with time, specifically letting
With $\gamma(t) = \gamma\_0 \exp(-\phi t)$ so that our model is

\begin{align}
\frac{\text{d}M}{\text{d}t} = & \gamma(t) S(t) - \lambda M(t) \\
\frac{\text{d}\rho\_{\text{M}}}{\text{d}t} = & \frac{\gamma(t) S(t)}{M(t)} \left( \rho\_\text{S}(t) - \rho\_\text{M}(t) \right).
\end{align}

For the resistant memory model we postulate that there is a memory sub-population which is self renewing and stable in number, with the number of these incumbent cells denoted $M\_{\text{inc}}$. In this case our model is

\begin{align}
\frac{\text{d}M}{\text{d}t} = & \gamma S(t) - \lambda (M(t) - M\_{\text{inc}} \\
\frac{\text{d}\rho\_{\text{M}}}{\text{d}t} = & \frac{\gamma S(t)}{M(t)} \left( \rho\_\text{S}(t) - \rho\_\text{M}(t) \right) - \frac{\lambda M\_{\text{inc}}}{M} \rho\_{\text{M}}(t).
\end{align}

When fitting our models it is important to remember that our Chimerism Spline expects the days post bone marrow transplant as an input, whereas the Count Spline expects the age of the mouse. We will run our ode models with days post BMT as our base time, and then transform appropriately for the age, i.e. $\text{age} = \text{age at BMT} + \text{days post BMT}$.

In [38]:

```
# the declining recruitment model
def dec_derivatives(y, dpBMT, params, SourceT_params, SourceCHI_params, ageBMT):
    T_t, CHI_t = y # Total cell counts and chimerism in target population
    gam, phi, lam = params # gamma_0, phi (decay of inflow) and lambda (net loss from M)
    #source counts take age = age at bmt + days post bmt as argument
    SourceT_t = exp_model(SourceT_params, ageBMT+dpBMT) 
    #source chimerism though just takes days post bmt
    SourceCHI_t = logistic_model(SourceCHI_params, dpBMT)
    # the parameters of gam(t) are going to be fit so they could take either
    # age or days post BMT, lets go with age, since we do expect this to be a
    # chimerism independent thing.
    gam_t = exp_model((gam, phi), ageBMT+dpBMT)
    dT_dt = gam_t * SourceT_t - lam * T_t
    dCHI_dt = gam_t * (SourceT_t / T_t) * (SourceCHI_t - CHI_t)
    der_array = np.array([dT_dt, dCHI_dt])
    return der_array
```

In [39]:

```
# wrap odeint for the declining recruitment model 
# to make it a little less cumbersome
def dec_ODE_model(params, dpBMT, T_0, CHI_0, SourceT_params, SourceCHI_params, ageBMT):
    gam, phi, lam = params
    smoothdpBMT = np.arange(np.min(dpBMT), np.max(dpBMT)+1)
    M = odeint(dec_derivatives, [T_0, CHI_0], smoothdpBMT, 
               args=([gam, phi, lam], SourceT_params, SourceCHI_params, ageBMT,))
    T = np.zeros((len(dpBMT)))
    CHI = np.zeros((len(dpBMT)))
    for ii,tt in enumerate(dpBMT):
        indx = tt==smoothdpBMT
        T[ii] = M[indx,0]
        CHI[ii] = M[indx,1]
    #M is at all of smoothdpBMT but T and CHI are nicely lined up 
    # with whatever crazy t vector is passed in
    return M, T, CHI
```

In [40]:

```
# the resistent memory (or incumbent) model
def inc_derivatives(y, dpBMT, params, SourceT_params, SourceCHI_params, ageBMT):
    T_t, CHI_t = y # Total cell counts and chimerism in memory
    gam, lam, Mi = params # gamma, lambda and resistant memory cells
    # Source counts take age as their time argument
    SourceT_t = exp_model(SourceT_params, ageBMT+dpBMT)
    # Source chimerism takes days post BMT as its time argument
    SourceCHI_t = logistic_model(SourceCHI_params, dpBMT)
    dT_dt = gam * SourceT_t - lam * (T_t - Mi)
    dCHI_dt = gam * (SourceT_t / T_t) * (SourceCHI_t - CHI_t) - lam * CHI_t * Mi/T_t
    der_array = np.array([dT_dt, dCHI_dt])
    return der_array
```

In [41]:

```
# wrap odeint for the imortal incumbent model to
# make it a little less cumbersome
def inc_ODE_model(params, dpBMT, T_0, CHI_0, SourceT_params, SourceCHI_params, ageBMT):
    gam, lam, Mi = params
    smoothdpBMT = np.arange(np.min(dpBMT), np.max(dpBMT)+1)
    M = odeint(inc_derivatives, [T_0, CHI_0], smoothdpBMT, 
               args=([gam, lam, Mi], SourceT_params, SourceCHI_params, ageBMT))
    T = np.zeros((len(dpBMT)))
    CHI = np.zeros((len(dpBMT)))
    for ii,tt in enumerate(dpBMT):
        indx = tt==smoothdpBMT
        T[ii] = M[indx,0]
        CHI[ii] = M[indx,1]
    #M is at all of smoothdpBMT but T and CHI are nicely lined up 
    # with whatever crazy t vector is passed in
    return M, T, CHI
```

We are also interested in the "weekly turnover". This is defined as the precentage of the compartment that are newly arrived since time $t\_0$ some amount of time $\tau$ later.

For the declining recruitment model the dynamics of "old" and "new" cells are given by
$$ \dot{M}\_{\text{old}} = - \lambda M\_{\text{old}}(t) $$
and
$$ \dot{M}\_{\text{new}} = \gamma(t) S(t) - \lambda M\_{new}(t). $$
Now, $S(t)$ is modeled as $S(t) = S\_0 \exp(-r t)$ and $\gamma(t)$ is modeled as $\gamma(t) = \gamma\_0 \exp(- \phi t)$ so this system of ODE's has an analytic solution, specifically.
$$ M\_{\text{old}}(\tau) = M(t) \exp(-\lambda \tau)$$
$$ M\_{\text{new}}(\tau) = \frac{\gamma(t) S(t)}{(\phi + r) - \lambda} (\exp(- \lambda \tau) - \exp(-(\phi + r) \tau))$$

For the resistent memory model the dynamic of "old" and "new" cells is given by
\begin{align}
\dot{M}*{\text{old}} = & - \lambda (M*{\text{old}}(t) - M*{i}) \
\dot{M}*{new} = & \gamma N(t) - \lambda M\_{\text{new}}(t).
\end{align}

Again, as $S(t)$ is modeled as $S(t) = S\_0 \exp(-r t)$ this system of ODE's has analytic solution
\begin{align}
M\_{\text{old}}(\tau) =& (M(t) - M\_i) \exp(-\lambda \tau) + M*i \
M*{\text{new}}(\tau) =& \frac{\gamma S(t)}{r - \lambda} (\exp(- \lambda \tau) - \exp(- r \tau))
\end{align}

Since the incumbent cells are not replaceable, it might also be nice to have a sense of how much of the population that could concievably turn over does in a given week. In which case dynamics of $M\_{old}$ would simply be as in the declinging recruitment model,
\begin{align}
M\_{\text{old\_replaceable}}(\tau) =& (M(t) - M\_i) \exp(-\lambda \tau) \
\end{align}

In [42]:

```
def dec_turnover(SourceT_smooth, T_smooth, target_params, SourceT_params, age, t_later):
    # here we compute the proportion of the memory pool that will be newly arived some time t_later from the current
    # moment given the parameters for SourceT and MT
    T_0, CHI_0, gam, phi, lam = target_params
    a, b = SourceT_params
    gam_t = exp_model((gam, phi), age)
    T_old = T_smooth * np.exp(- lam * t_later)
    T_new = gam_t * SourceT_smooth / ((phi + b) - lam) * (np.exp(-lam * t_later) - np.exp(-(phi + b) * t_later))
    turnover = T_new / (T_old + T_new)
    return turnover
```

In [43]:

```
def inc_turnover(SourceT_smooth, T_smooth, target_params, SourceT_params, t_later):
    # here we compute the proportion of the memory pool that will be newly arived some 
    # time t_later from the current moment given the parameters for SourceT and MT
    T_0, CHI_0, gam, lam, Mi = target_params
    a, b = SourceT_params
    T_old = (T_smooth - Mi) * np.exp(-lam * t_later) + Mi
    T_new = gam * SourceT_smooth / (b - lam) * (np.exp(-lam * t_later) - np.exp(-b * t_later))
    turnover = T_new / (T_old + T_new)
    return turnover
```

In [44]:

```
def inc_turnover_no_inc(SourceT_smooth, T_smooth, target_params, SourceT_params, t_later):
    # here we compute the proportion of the memory pool
    # that can be replaced, that will be newly arived some 
    # time t_later from the current moment given the parameters for SourceT and MT
    T_0, CHI_0, gam, lam, Mi = target_params
    a, b = SourceT_params
    # same as above but without the incumbents counted
    T_old = (T_smooth - Mi) * np.exp(-lam * t_later)
    T_new = gam * SourceT_smooth / (b - lam) * (np.exp(-lam * t_later) - np.exp(-b * t_later))
    turnover = T_new / (T_old + T_new)
    return turnover
```

Here we have two cruves to fit, both determined by shared parameters. We will use a maximum likelihood approach to fitting these data. Under some assumptions about the normality of residuals, homoscadsticity, and uncorrelated errors, which are roughly met, the likelihood is the the product of the SSE for the chimerism curve and the SSE for the counts curve. Rather than simply plugging the product of the SSE of both curves into some general optimizer, we take advantage of non-linear least squares methods (more stable and sometimes faster because they take advantage of the fact that the top layer of the cost function is the sum of some squared terms). Thus we scale the residuals from each curve by the square root of the SSE of the other curve, this lets us continue to take advantage of the locally quadratic shape of our cost function even though it is actually the product of the sum of squared errors and not just a simple sum of squared errors.

In [45]:

```
# we transform some of the raw parameters so that the 
# non linear least squares optimizer has unbounded parameters
# to work with.
def dec_raw_params_to_trans(raw_params):
    T_0, CHI_0, gam, phi, lam = raw_params
    log_T_0 = np.log(T_0) 
    log_CHI_0 = np.log(CHI_0)
    log_gam = np.log(gam)
    trans_params = [log_T_0, log_CHI_0, log_gam, phi, lam]
    return trans_params

def dec_trans_params_to_raw(trans_params):
    log_T_0, log_CHI_0, log_gam, phi, lam = trans_params
    T_0 = np.exp(log_T_0)
    CHI_0 = np.exp(log_CHI_0)
    gam = np.exp(log_gam)
    raw_params = [T_0, CHI_0, gam, phi, lam]
    return raw_params
    
def dec_cross_residuals_ODE_trans(trans_params, dpBMT, log_T_obs, logit_CHI_obs, 
                                  SourceT_params, SourceCHI_params, ageBMT):
    raw_params = dec_trans_params_to_raw(trans_params)
    T_0, CHI_0, gam, phi, lam = raw_params
    M_model, T_model, CHI_model = dec_ODE_model([gam, phi, lam], dpBMT, T_0, CHI_0, 
                                                SourceT_params, SourceCHI_params, ageBMT)
    T_log_residuals = log_T_obs - np.log(T_model)
    CHI_logit_residuals = logit_CHI_obs - logit(CHI_model)
    T_log_SSE = np.sum(T_log_residuals**2)
    CHI_logit_SSE = np.sum(CHI_logit_residuals**2)
    T_cross_scaled_log_residuals = T_log_residuals * np.sqrt(CHI_logit_SSE)
    CHI_cross_scaled_logit_residuals = CHI_logit_residuals * np.sqrt(T_log_SSE)
    total_cross_scaled_residuals = np.concatenate((T_cross_scaled_log_residuals, CHI_cross_scaled_logit_residuals))
    return total_cross_scaled_residuals
```

In [46]:

```
# similarly we transform the raw params so the non-linear
# least squares optimizer has undbounded parameters to work with
def inc_raw_params_to_trans(raw_params):
    T_0, CHI_0, gam, lam, Mi = raw_params
    log_T_0_less_Mi = np.log(T_0 - Mi) 
    log_CHI_0 = np.log(CHI_0)
    log_gam = np.log(gam)
    log_Mi = np.log(Mi)
    trans_params = [log_T_0_less_Mi, log_CHI_0, log_gam, lam, log_Mi]
    return trans_params

def inc_trans_params_to_raw(trans_params):
    log_T_0_less_Mi, log_CHI_0, log_gam, lam, log_Mi = trans_params
    Mi = np.exp(log_Mi)
    T_0 = np.exp(log_T_0_less_Mi) + Mi
    CHI_0 = np.exp(log_CHI_0)
    gam = np.exp(log_gam)
    raw_params = [T_0, CHI_0, gam, lam, Mi]
    return raw_params
    
def inc_cross_residuals_ODE_trans(trans_params, time, log_T_obs, logit_CHI_obs, 
                                  SourceT_params, SourceCHI_params, ageBMT):
    raw_params = inc_trans_params_to_raw(trans_params)
    T_0, CHI_0, gam, lam, Mi = raw_params
    M_model, T_model, CHI_model = inc_ODE_model([gam, lam, Mi], time, T_0, CHI_0, 
                                                SourceT_params, SourceCHI_params, ageBMT)
    T_log_residuals = log_T_obs - np.log(T_model)
    CHI_logit_residuals = logit_CHI_obs - logit(CHI_model)
    T_log_SSE = np.sum(T_log_residuals**2)
    CHI_logit_SSE = np.sum(CHI_logit_residuals**2)
    T_cross_scaled_log_residuals = T_log_residuals * np.sqrt(CHI_logit_SSE)
    CHI_cross_scaled_logit_residuals = CHI_logit_residuals * np.sqrt(T_log_SSE)
    total_cross_scaled_residuals = np.concatenate((T_cross_scaled_log_residuals, CHI_cross_scaled_logit_residuals))
    return total_cross_scaled_residuals
```

In [47]:

```
def fit_T_CHI(model,
              T_obs, CHI_obs, log_T_obs, logit_CHI_obs, 
              SourceT_params, SourceCHI_params, param_guess, 
              dpBMT, ageBMT,
              log_T_res=1, logit_CHI_res=1):
    # T_0, CHI_0, and if applicable Mi we guess straight from the data,
    # the other paramaters gam, lam and where apllicable phi are passed in via param_guess
    init_dpBMT_indices = dpBMT == np.min(dpBMT)
    T_0_guess = np.mean(T_obs[init_dpBMT_indices])
    CHI_0_guess = np.mean(CHI_obs[init_dpBMT_indices])
    # we set up the fit so that it fits bootstraps by resampling residuals 
    # if residuals are passed in otherwise it just fits to the data
    np.random.seed()
    fake_log_T_obs = log_T_obs + np.random.choice(log_T_res, len(log_T_obs))
    fake_logit_CHI_obs = logit_CHI_obs + np.random.choice(logit_CHI_res, len(logit_CHI_obs))
    ls_args = (dpBMT, fake_log_T_obs, fake_logit_CHI_obs, 
               SourceT_params, SourceCHI_params, ageBMT)
    if model == 'inc':
        Mi_guess = 0.5 * np.mean(T_obs[init_dpBMT_indices])
        params_guess = [T_0_guess, CHI_0_guess] + param_guess + [Mi_guess]
        trans_params_guess = inc_raw_params_to_trans(params_guess)
        result = least_squares(inc_cross_residuals_ODE_trans, trans_params_guess, 
                               method='trf', loss='linear', args=ls_args)
        trans_params_hat = result['x']
        raw_params_hat = inc_trans_params_to_raw(trans_params_hat) 
    elif model == 'dec':
        params_guess = [T_0_guess, CHI_0_guess] + param_guess
        trans_params_guess = dec_raw_params_to_trans(params_guess)
        result = least_squares(dec_cross_residuals_ODE_trans, trans_params_guess, 
                               method='trf', loss='linear', args=ls_args)
        trans_params_hat = result['x']
        raw_params_hat = dec_trans_params_to_raw(trans_params_hat) 
    cost = result['cost']
    return raw_params_hat, cost
```

In [48]:

```
def make_curves_from_params(model, target_params, SourceT_params, 
                            SourceCHI_params, dpBMT, ageBMT):
    smoothdpBMT = np.arange(np.min(dpBMT), np.max(dpBMT)+1)
    SourceT_smooth = exp_model(SourceT_params, smoothdpBMT+ageBMT)
    if model == 'dec':
        T_0, CHI_0, gam, phi, lam = target_params
        ode_args = ([gam, phi, lam], SourceT_params, SourceCHI_params, ageBMT)
        M = odeint(dec_derivatives, [T_0, CHI_0], smoothdpBMT, args=ode_args)
    elif model == 'inc':
        T_0, CHI_0, gam, lam, Mi = target_params
        ode_args = ([gam, lam, Mi], SourceT_params, SourceCHI_params, ageBMT)
        M = odeint(inc_derivatives, [T_0, CHI_0], smoothdpBMT, args=ode_args)
    # our smooth curves
    T_smooth = M[:,0]
    CHI_smooth = M[:,1]
    if model == 'dec':
        gam_t_smooth = exp_model((gam, phi), ageBMT+smoothdpBMT)
        In = gam_t_smooth * SourceT_smooth / T_smooth
        Turn = dec_turnover(SourceT_smooth, T_smooth, target_params, 
                            SourceT_params, smoothdpBMT+ageBMT, 7)
        Turn_no_inc = Turn
        Inc_prop = np.zeros(len(Turn))
    elif model == 'inc':
        In = gam * SourceT_smooth / T_smooth
        Turn = inc_turnover(SourceT_smooth, T_smooth, target_params, 
                            SourceT_params, 7)
        Turn_no_inc = inc_turnover_no_inc(SourceT_smooth, T_smooth, target_params, 
                                          SourceT_params, 7)
        Inc_prop = Mi / np.array(T_smooth)
    curves = [T_smooth, CHI_smooth, In, Turn, Turn_no_inc, Inc_prop]
    # our predictions that line up with the data
    T = np.zeros((len(dpBMT)))
    CHI = np.zeros((len(dpBMT)))
    for ii,tt in enumerate(dpBMT):
        indx = tt==smoothdpBMT
        T[ii] = T_smooth[indx]
        CHI[ii] = CHI_smooth[indx]
    predictions = [T, CHI]
    return predictions, curves
```

In [49]:

```
def make_residuals(T, log_T, CHI, logit_CHI, T_hat, CHI_hat):
    T_residuals = T - T_hat
    T_log_residuals = log_T - np.log(T_hat)
    CHI_residuals = CHI - CHI_hat
    CHI_logit_residuals = logit_CHI - logit(CHI_hat)
    T_log_SSE = np.sum(T_log_residuals**2)
    CHI_logit_SSE = np.sum(CHI_logit_residuals**2)
    cost = T_log_SSE * CHI_logit_SSE
    neg_log_Lik = len(T)*(np.log(T_log_SSE) + np.log(CHI_logit_SSE))
    AIC = 2 * (neg_log_Lik + (5*len(T)/(len(T)-5-1)))
    BIC = 2 * (neg_log_Lik) + np.log(len(T))*5.0
    return T_residuals, T_log_residuals, CHI_residuals, CHI_logit_residuals, cost, neg_log_Lik, AIC, BIC
```

In [50]:

```
def make_figs_of_fits(T, CHI, T_hat_smooth, CHI_hat_smooth, dpBMT, ageBMT, title):
    smoothdpBMT = np.arange(np.min(dpBMT), np.max(dpBMT)+1)
    figT, axT = plt.subplots()
    axT.scatter(dpBMT, T)
    axT.plot(smoothdpBMT, T_hat_smooth)
    axT.set_xlabel('days post bone marrow transplant')
    axT.set_ylabel('cell counts')
    axT2 = axT.twiny()
    axT2.set_xlim(axT.get_xlim())
    axT2.set_xticklabels((axT.get_xticks() + ageBMT).astype(int))
    axT2.set_xlabel('mouse age in days')
    axT2.set_title('Counts: ' + title, y=1.1)
    
    figCHI, axCHI = plt.subplots()
    axCHI.scatter(dpBMT, CHI)
    axCHI.plot(smoothdpBMT, CHI_hat_smooth)
    axCHI.set_xlabel('days post bone marrow transplant')
    axCHI.set_ylabel('Normalized Chimerism')
    axCHI2 = axCHI.twiny()
    axCHI2.set_xlim(axCHI.get_xlim())
    axCHI2.set_xticklabels((axCHI.get_xticks() + ageBMT).astype(int))
    axCHI2.set_xlabel('mouse age in days')
    axCHI2.set_title('Chimerism: ' + title, y=1.1)
```

In [51]:

```
def do_model_analysis(model = 'dec', 
                      sourceT_params = NT_params_hat, 
                      sourceCHI_params = NCHI_params_hat, 
                      target = '4Tem', 
                      logT_res=1, 
                      logitCHI_res=1,
                      params_guess = None,
                      verbose=True, 
                      title=''):
    if model == 'dec':
        if params_guess is None:
            params_guess = [0.001, -0.001, 0.001] # gam, phi, lam
    elif model == 'inc':
        if params_guess is None:
            params_guess = [0.01, 0.01] # gam, lam
    else:
        print('invalid model choice, should be inc or dec')
    if not target in ['4Tem', '4Tcm']:
        print('invalid choice of target should be 4Tem or 4Tcm')
    T = ddTarget['TOTAL.'+target]
    CHI = ddTarget['NCHI.'+target]
    logT = ddTarget['log.TOTAL.'+target]
    logitCHI = ddTarget['logit.NCHI.'+target]
    dpBMT = ddTarget['days.post.bmt']
    ageBMT = ddTarget['age.at.bmt'].values[0]
    # age at BMT should be 56 for all of these mice

    params_hat, cost1 = fit_T_CHI(model, T, CHI, logT, logitCHI,
                                  sourceT_params, sourceCHI_params,
                                  params_guess, dpBMT, ageBMT,
                                  log_T_res=logT_res, logit_CHI_res=logitCHI_res) 
    predictions, curves = make_curves_from_params(model, params_hat, 
                                                  sourceT_params, sourceCHI_params,
                                                  dpBMT, ageBMT)
    residuals_etc = 0
    if verbose:
        if model == 'dec':
            print('The mle parameters are (T_0, CHI_0, gam, phi, lam):')
        elif model == 'inc':
            print('The mle parameters are (T_0, CHI_0, gam, lam, Mi):')
        print(params_hat)
        
        residuals_etc = make_residuals(T, logT, CHI, logitCHI, 
                                       predictions[0], predictions[1])
        (T_residuals, logT_residuals, 
         CHI_residuals, logitCHI_residuals,
         cost2, neg_logLik, AIC, BIC) = residuals_etc
        print('AIC for this model is:')
        print(AIC)
        print('BIC for this model is:')
        print(BIC)
        make_figs_of_fits(T, CHI, curves[0], curves[1], dpBMT, ageBMT, title)
    return params_hat, curves, residuals_etc
```

In [52]:

```
title='Effector Memory with Naive as source, declining recruitment'
(dec_EN_params_hat, 
 dec_EN_curves, 
 dec_EN_residuals_etc) = do_model_analysis('dec', NT_params_hat, NCHI_params_hat, 
                                          '4Tem', title = title)
```

```
The mle parameters are (T_0, CHI_0, gam, phi, lam):
[5550481.6700599194, 0.025582720928191138, 0.0022005605811038094, 0.0021623315454434222, 0.00022325321336952558]
AIC for this model is:
110.538362702
BIC for this model is:
113.09529852
```

In [53]:

```
title = 'Effector Memory with Naive as source, resistant memory'
(inc_EN_params_hat, 
 inc_EN_curves, 
 inc_EN_residuals_etc) = do_model_analysis('inc', NT_params_hat, NCHI_params_hat, 
                                          '4Tem', title = title)
```

```
The mle parameters are (T_0, CHI_0, gam, lam, Mi):
[5515870.7129528448, 0.02570546895803744, 0.0017246706421891621, 0.0015089448546917496, 5333831.5373881208]
AIC for this model is:
110.698116635
BIC for this model is:
113.255052454
```

In [54]:

```
title = 'Effector Memory with Central Memory as source, declining recruitment'
(dec_EC_params_hat, 
 dec_EC_curves, 
 dec_EC_residuals_etc) = do_model_analysis('dec', CT_spline_params_hat, 
                                           CCHI_spline_params_hat, 
                                          '4Tem', title = title)
```

```
The mle parameters are (T_0, CHI_0, gam, phi, lam):
[4424272.9903185265, 0.0274753547470762, 0.17242212878519816, -0.00082220681669736384, 0.010888518304446353]
AIC for this model is:
121.877299065
BIC for this model is:
124.434234884
```

In [55]:

```
title = 'Effector Memory with Central Memory as source, resistant'
(inc_EC_params_hat, 
 inc_EC_curves, 
 inc_EC_residuals_etc) = do_model_analysis('inc', CT_spline_params_hat, 
                                           CCHI_spline_params_hat, 
                                          '4Tem', title = title)
```

```
The mle parameters are (T_0, CHI_0, gam, lam, Mi):
[4250902.856793182, 0.027229437809725549, 0.20293896011404683, 0.011542739758739819, 616945.32888456504]
AIC for this model is:
121.80420002
BIC for this model is:
124.361135838
```

In [56]:

```
title = 'Central Memory with Naive as Source, declining recruitment'
(dec_CN_params_hat, 
 dec_CN_curves, 
 dec_CN_residuals_etc) = do_model_analysis('dec', NT_params_hat, 
                                           NCHI_params_hat, 
                                          '4Tcm', title = title)
```

```
The mle parameters are (T_0, CHI_0, gam, phi, lam):
[689128.5942129239, 0.072587430312761284, 0.0013194690161799202, 0.0097006853675301596, 0.0071492036332446059]
AIC for this model is:
150.868769718
BIC for this model is:
153.425705537
```

In [57]:

```
title = 'Central Memory with Naive as Source, resistant memory'
(inc_CN_params_hat, 
 inc_CN_curves, 
 inc_CN_residuals_etc) = do_model_analysis('inc', NT_params_hat, 
                                           NCHI_params_hat, 
                                          '4Tcm', title = title)
```

```
The mle parameters are (T_0, CHI_0, gam, lam, Mi):
[926743.51294126827, 0.074153402737072202, 0.00045226493215857425, 0.019060539041176303, 145532.92550442301]
AIC for this model is:
142.591010743
BIC for this model is:
145.147946561
```

In [58]:

```
dfAIC = pd.DataFrame()
dfAIC['model'] = ['dec_CN', 'inc_CN', 'dec_EN', 'inc_EN', 'dec_EC', 'inc_EC']
dfAIC['AIC'] = [eval(x+'_residuals_etc')[-2] for x in dfAIC['model'].values]
dfAIC['BIC'] = [eval(x+'_residuals_etc')[-1] for x in dfAIC['model'].values]
# modeltype_targetPopulation_sourcePopulation
dfAIC
```

Out[58]:

|  | model | AIC | BIC |
| --- | --- | --- | --- |
| 0 | dec\_CN | 150.868770 | 153.425706 |
| 1 | inc\_CN | 142.591011 | 145.147947 |
| 2 | dec\_EN | 110.538363 | 113.095299 |
| 3 | inc\_EN | 110.698117 | 113.255052 |
| 4 | dec\_EC | 121.877299 | 124.434235 |
| 5 | inc\_EC | 121.804200 | 124.361136 |

Now we get a sense of our confidence in our parameter estimates for these six different models using bootstraps. Note that we not only resample residuals for these bootstrap estimates, we also resample from our bootstrap distribution of the parameter estimates for the source curve. In this way we propagate our uncertainty about the source curves into our estimates of the parameters of the mechanistic ODE model.

In [59]:

```
num_bootstraps = 10000 # make this number smaller if you don't have lots of cores
inputs = range(num_bootstraps)
```

In [60]:

```
def inc_EN_boot_wrap(i):
    np.random.seed()
    fake_index = np.random.choice(len(NT_bootstrap_params[0,:]))
    # assume that we have the same number of bootstrap params for each spline 
    fake_NT_params = NT_bootstrap_params[:,fake_index]
    fake_NCHI_params = NCHI_bootstrap_params[:,fake_index]
    
    (inc_EN_params_boot,
     inc_EN_curves_boot,
     inc_EN_residuals_boot) = do_model_analysis('inc', fake_NT_params,
                                                fake_NCHI_params, '4Tem', 
                                                logT_res = inc_EN_residuals_etc[1],
                                                logitCHI_res = inc_EN_residuals_etc[3],
                                                params_guess = inc_EN_params_hat[2:-1],
                                                verbose=False)
    
    return (inc_EN_params_boot, inc_EN_curves_boot)

results = Parallel(n_jobs=num_cores, verbose=5)(delayed(inc_EN_boot_wrap)(i) for i in inputs)
inc_EN_bootstrap_params, inc_EN_bootstrap_curves = zip(*results)
# for historical reasons we have to do transposing and axes swapping so these
# object are the shape expected downstream
inc_EN_bootstrap_params = np.array(inc_EN_bootstrap_params).T
inc_EN_bootstrap_curves = np.swapaxes(np.array(inc_EN_bootstrap_curves), 0, 2)
```

```
[Parallel(n_jobs=56)]: Done  50 tasks      | elapsed:    2.4s
[Parallel(n_jobs=56)]: Done 176 tasks      | elapsed:    6.1s
[Parallel(n_jobs=56)]: Done 338 tasks      | elapsed:   12.0s
[Parallel(n_jobs=56)]: Done 536 tasks      | elapsed:   19.3s
[Parallel(n_jobs=56)]: Done 770 tasks      | elapsed:   27.7s
[Parallel(n_jobs=56)]: Done 1040 tasks      | elapsed:   37.2s
[Parallel(n_jobs=56)]: Done 1346 tasks      | elapsed:   47.8s
[Parallel(n_jobs=56)]: Done 1688 tasks      | elapsed:  1.0min
[Parallel(n_jobs=56)]: Done 2066 tasks      | elapsed:  1.2min
[Parallel(n_jobs=56)]: Done 2480 tasks      | elapsed:  1.5min
[Parallel(n_jobs=56)]: Done 2930 tasks      | elapsed:  1.7min
[Parallel(n_jobs=56)]: Done 3416 tasks      | elapsed:  2.0min
[Parallel(n_jobs=56)]: Done 3938 tasks      | elapsed:  2.3min
[Parallel(n_jobs=56)]: Done 4496 tasks      | elapsed:  2.7min
[Parallel(n_jobs=56)]: Done 5090 tasks      | elapsed:  3.0min
[Parallel(n_jobs=56)]: Done 5720 tasks      | elapsed:  3.4min
[Parallel(n_jobs=56)]: Done 6386 tasks      | elapsed:  3.7min
[Parallel(n_jobs=56)]: Done 7088 tasks      | elapsed:  4.1min
[Parallel(n_jobs=56)]: Done 7826 tasks      | elapsed:  4.6min
[Parallel(n_jobs=56)]: Done 8600 tasks      | elapsed:  5.0min
[Parallel(n_jobs=56)]: Done 9410 tasks      | elapsed:  5.5min
[Parallel(n_jobs=56)]: Done 10000 out of 10000 | elapsed:  5.9min finished
```

In [61]:

```
def inc_EC_boot_wrap(i):
    np.random.seed()
    fake_index = np.random.choice(len(CT_spline_bootstrap_params[0,:]))
    # assume that we have the same number of bootstrap params for each spline 
    fake_CT_params = CT_spline_bootstrap_params[:,fake_index]
    fake_CCHI_params = CCHI_spline_bootstrap_params[:,fake_index]
    
    (inc_EC_params_boot,
     inc_EC_curves_boot,
     inc_EC_residuals_boot) = do_model_analysis('inc', fake_CT_params,
                                                fake_CCHI_params, '4Tem', 
                                                logT_res = inc_EC_residuals_etc[1],
                                                logitCHI_res = inc_EC_residuals_etc[3],
                                                params_guess = inc_EC_params_hat[2:-1],
                                                verbose=False)
    
    return (inc_EC_params_boot, inc_EC_curves_boot)

results = Parallel(n_jobs=num_cores, verbose=5)(delayed(inc_EC_boot_wrap)(i) for i in inputs)
inc_EC_bootstrap_params, inc_EC_bootstrap_curves = zip(*results)
# for historical reasons we have to do transposing and axes swapping so these
# object are the shape expected downstream
inc_EC_bootstrap_params = np.array(inc_EC_bootstrap_params).T
inc_EC_bootstrap_curves = np.swapaxes(np.array(inc_EC_bootstrap_curves), 0, 2)
```

```
[Parallel(n_jobs=56)]: Done  50 tasks      | elapsed:    2.4s
[Parallel(n_jobs=56)]: Done 176 tasks      | elapsed:    6.5s
[Parallel(n_jobs=56)]: Done 338 tasks      | elapsed:   11.9s
[Parallel(n_jobs=56)]: Done 536 tasks      | elapsed:   18.9s
/home/dcownden/anaconda2/envs/py3/lib/python3.5/site-packages/ipykernel/__main__.py:3: RuntimeWarning: invalid value encountered in log
  app.launch_new_instance()
[Parallel(n_jobs=56)]: Done 770 tasks      | elapsed:   26.6s
[Parallel(n_jobs=56)]: Done 1040 tasks      | elapsed:   34.7s
[Parallel(n_jobs=56)]: Done 1346 tasks      | elapsed:   44.2s
[Parallel(n_jobs=56)]: Done 1688 tasks      | elapsed:   55.2s
[Parallel(n_jobs=56)]: Done 2066 tasks      | elapsed:  1.1min
/home/dcownden/anaconda2/envs/py3/lib/python3.5/site-packages/scipy/integrate/odepack.py:218: ODEintWarning: Excess work done on this call (perhaps wrong Dfun type). Run with full_output = 1 to get quantitative information.
  warnings.warn(warning_msg, ODEintWarning)
/home/dcownden/anaconda2/envs/py3/lib/python3.5/site-packages/ipykernel/__main__.py:3: RuntimeWarning: invalid value encountered in log
  app.launch_new_instance()
/home/dcownden/anaconda2/envs/py3/lib/python3.5/site-packages/scipy/integrate/odepack.py:218: ODEintWarning: Excess work done on this call (perhaps wrong Dfun type). Run with full_output = 1 to get quantitative information.
  warnings.warn(warning_msg, ODEintWarning)
/home/dcownden/anaconda2/envs/py3/lib/python3.5/site-packages/ipykernel/__main__.py:3: RuntimeWarning: invalid value encountered in log
  app.launch_new_instance()
[Parallel(n_jobs=56)]: Done 2480 tasks      | elapsed:  1.4min
/home/dcownden/anaconda2/envs/py3/lib/python3.5/site-packages/ipykernel/__main__.py:3: RuntimeWarning: invalid value encountered in log
  app.launch_new_instance()
[Parallel(n_jobs=56)]: Done 2930 tasks      | elapsed:  1.6min
[Parallel(n_jobs=56)]: Done 3416 tasks      | elapsed:  1.8min
/home/dcownden/anaconda2/envs/py3/lib/python3.5/site-packages/ipykernel/__main__.py:3: RuntimeWarning: invalid value encountered in log
  app.launch_new_instance()
[Parallel(n_jobs=56)]: Done 3938 tasks      | elapsed:  2.1min
/home/dcownden/anaconda2/envs/py3/lib/python3.5/site-packages/ipykernel/__main__.py:3: RuntimeWarning: invalid value encountered in log
  app.launch_new_instance()
[Parallel(n_jobs=56)]: Done 4496 tasks      | elapsed:  2.4min
/home/dcownden/anaconda2/envs/py3/lib/python3.5/site-packages/ipykernel/__main__.py:3: RuntimeWarning: invalid value encountered in log
  app.launch_new_instance()
[Parallel(n_jobs=56)]: Done 5090 tasks      | elapsed:  2.7min
/home/dcownden/anaconda2/envs/py3/lib/python3.5/site-packages/ipykernel/__main__.py:3: RuntimeWarning: invalid value encountered in log
  app.launch_new_instance()
[Parallel(n_jobs=56)]: Done 5720 tasks      | elapsed:  3.0min
[Parallel(n_jobs=56)]: Done 6386 tasks      | elapsed:  3.4min
[Parallel(n_jobs=56)]: Done 7088 tasks      | elapsed:  3.7min
[Parallel(n_jobs=56)]: Done 7826 tasks      | elapsed:  4.1min
[Parallel(n_jobs=56)]: Done 8600 tasks      | elapsed:  4.5min
[Parallel(n_jobs=56)]: Done 9410 tasks      | elapsed:  4.9min
[Parallel(n_jobs=56)]: Done 10000 out of 10000 | elapsed:  5.2min finished
```

In [62]:

```
def inc_CN_boot_wrap(i):
    np.random.seed()
    fake_index = np.random.choice(len(NT_bootstrap_params[0,:]))
    # assume that we have the same number of bootstrap params for each spline 
    fake_NT_params = NT_bootstrap_params[:,fake_index]
    fake_NCHI_params = NCHI_bootstrap_params[:,fake_index]
    
    (inc_CN_params_boot,
     inc_CN_curves_boot,
     inc_CN_residuals_boot) = do_model_analysis('inc', fake_NT_params,
                                                fake_NCHI_params, '4Tcm', 
                                                logT_res = inc_CN_residuals_etc[1],
                                                logitCHI_res = inc_CN_residuals_etc[3],
                                                params_guess = inc_CN_params_hat[2:-1],
                                                verbose=False)
    
    return (inc_CN_params_boot, inc_CN_curves_boot)

results = Parallel(n_jobs=num_cores, verbose=5)(delayed(inc_CN_boot_wrap)(i) for i in inputs)
inc_CN_bootstrap_params, inc_CN_bootstrap_curves = zip(*results)
# for historical reasons we have to do transposing and axes swapping so these
# object are the shape expected downstream
inc_CN_bootstrap_params = np.array(inc_CN_bootstrap_params).T
inc_CN_bootstrap_curves = np.swapaxes(np.array(inc_CN_bootstrap_curves), 0, 2)
```

```
[Parallel(n_jobs=56)]: Done  50 tasks      | elapsed:    1.7s
[Parallel(n_jobs=56)]: Done 176 tasks      | elapsed:    4.5s
[Parallel(n_jobs=56)]: Done 338 tasks      | elapsed:    8.1s
[Parallel(n_jobs=56)]: Done 536 tasks      | elapsed:   12.8s
[Parallel(n_jobs=56)]: Done 770 tasks      | elapsed:   17.9s
[Parallel(n_jobs=56)]: Done 1040 tasks      | elapsed:   23.5s
[Parallel(n_jobs=56)]: Done 1346 tasks      | elapsed:   30.3s
[Parallel(n_jobs=56)]: Done 1688 tasks      | elapsed:   37.0s
[Parallel(n_jobs=56)]: Done 2066 tasks      | elapsed:   44.5s
[Parallel(n_jobs=56)]: Done 2480 tasks      | elapsed:   53.9s
[Parallel(n_jobs=56)]: Done 2930 tasks      | elapsed:  1.1min
[Parallel(n_jobs=56)]: Done 3416 tasks      | elapsed:  1.2min
[Parallel(n_jobs=56)]: Done 3938 tasks      | elapsed:  1.4min
[Parallel(n_jobs=56)]: Done 4496 tasks      | elapsed:  1.6min
[Parallel(n_jobs=56)]: Done 5090 tasks      | elapsed:  1.8min
[Parallel(n_jobs=56)]: Done 5720 tasks      | elapsed:  2.0min
[Parallel(n_jobs=56)]: Done 6386 tasks      | elapsed:  2.2min
[Parallel(n_jobs=56)]: Done 7088 tasks      | elapsed:  2.5min
[Parallel(n_jobs=56)]: Done 7826 tasks      | elapsed:  2.7min
[Parallel(n_jobs=56)]: Done 8600 tasks      | elapsed:  3.0min
[Parallel(n_jobs=56)]: Done 9410 tasks      | elapsed:  3.3min
[Parallel(n_jobs=56)]: Done 10000 out of 10000 | elapsed:  3.5min finished
```

In [63]:

```
def dec_EN_boot_wrap(i):
    np.random.seed()
    fake_index = np.random.choice(len(NT_bootstrap_params[0,:]))
    # assume that we have the same number of bootstrap params for each spline 
    fake_NT_params = NT_bootstrap_params[:,fake_index]
    fake_NCHI_params = NCHI_bootstrap_params[:,fake_index]
    
    (dec_EN_params_boot,
     dec_EN_curves_boot,
     dec_EN_residuals_boot) = do_model_analysis('dec', fake_NT_params,
                                                fake_NCHI_params, '4Tem', 
                                                logT_res = dec_EN_residuals_etc[1],
                                                logitCHI_res = dec_EN_residuals_etc[3],
                                                params_guess = dec_EN_params_hat[2:],
                                                verbose=False)
    
    return (dec_EN_params_boot, dec_EN_curves_boot)

results = Parallel(n_jobs=num_cores, verbose=5)(delayed(dec_EN_boot_wrap)(i) for i in inputs)
dec_EN_bootstrap_params, dec_EN_bootstrap_curves = zip(*results)
# for historical reasons we have to do transposing and axes swapping so these
# object are the shape expected downstream
dec_EN_bootstrap_params = np.array(dec_EN_bootstrap_params).T
dec_EN_bootstrap_curves = np.swapaxes(np.array(dec_EN_bootstrap_curves), 0, 2)
```

```
[Parallel(n_jobs=56)]: Done  50 tasks      | elapsed:    2.2s
[Parallel(n_jobs=56)]: Done 176 tasks      | elapsed:    6.5s
[Parallel(n_jobs=56)]: Done 338 tasks      | elapsed:   11.5s
[Parallel(n_jobs=56)]: Done 536 tasks      | elapsed:   17.8s
[Parallel(n_jobs=56)]: Done 770 tasks      | elapsed:   25.3s
[Parallel(n_jobs=56)]: Done 1040 tasks      | elapsed:   33.0s
[Parallel(n_jobs=56)]: Done 1346 tasks      | elapsed:   41.8s
[Parallel(n_jobs=56)]: Done 1688 tasks      | elapsed:   52.0s
[Parallel(n_jobs=56)]: Done 2066 tasks      | elapsed:  1.0min
[Parallel(n_jobs=56)]: Done 2480 tasks      | elapsed:  1.3min
[Parallel(n_jobs=56)]: Done 2930 tasks      | elapsed:  1.5min
[Parallel(n_jobs=56)]: Done 3416 tasks      | elapsed:  1.7min
[Parallel(n_jobs=56)]: Done 3938 tasks      | elapsed:  1.9min
[Parallel(n_jobs=56)]: Done 4496 tasks      | elapsed:  2.2min
[Parallel(n_jobs=56)]: Done 5090 tasks      | elapsed:  2.5min
[Parallel(n_jobs=56)]: Done 5720 tasks      | elapsed:  2.8min
[Parallel(n_jobs=56)]: Done 6386 tasks      | elapsed:  3.2min
[Parallel(n_jobs=56)]: Done 7088 tasks      | elapsed:  3.6min
[Parallel(n_jobs=56)]: Done 7826 tasks      | elapsed:  3.9min
[Parallel(n_jobs=56)]: Done 8600 tasks      | elapsed:  4.3min
[Parallel(n_jobs=56)]: Done 9410 tasks      | elapsed:  4.8min
[Parallel(n_jobs=56)]: Done 10000 out of 10000 | elapsed:  5.2min finished
```

In [64]:

```
def dec_EC_boot_wrap(i):
    np.random.seed()
    fake_index = np.random.choice(len(CT_spline_bootstrap_params[0,:]))
    # assume that we have the same number of bootstrap params for each spline 
    fake_CT_params = CT_spline_bootstrap_params[:,fake_index]
    fake_CCHI_params = CCHI_spline_bootstrap_params[:,fake_index]
    
    (dec_EC_params_boot,
     dec_EC_curves_boot,
     dec_EC_residuals_boot) = do_model_analysis('dec', fake_CT_params,
                                                fake_CCHI_params, '4Tem', 
                                                logT_res = dec_EC_residuals_etc[1],
                                                logitCHI_res = dec_EC_residuals_etc[3],
                                                params_guess = dec_EC_params_hat[2:],
                                                verbose=False)
    
    return (dec_EC_params_boot, dec_EC_curves_boot)

results = Parallel(n_jobs=num_cores, verbose=5)(delayed(dec_EC_boot_wrap)(i) for i in inputs)
dec_EC_bootstrap_params, dec_EC_bootstrap_curves = zip(*results)
# for historical reasons we have to do transposing and axes swapping so these
# object are the shape expected downstream
dec_EC_bootstrap_params = np.array(dec_EC_bootstrap_params).T
dec_EC_bootstrap_curves = np.swapaxes(np.array(dec_EC_bootstrap_curves), 0, 2)
```

```
[Parallel(n_jobs=56)]: Done  50 tasks      | elapsed:    2.4s
[Parallel(n_jobs=56)]: Done 176 tasks      | elapsed:    6.3s
[Parallel(n_jobs=56)]: Done 338 tasks      | elapsed:   11.8s
[Parallel(n_jobs=56)]: Done 536 tasks      | elapsed:   19.4s
[Parallel(n_jobs=56)]: Done 770 tasks      | elapsed:   27.6s
[Parallel(n_jobs=56)]: Done 1040 tasks      | elapsed:   38.6s
[Parallel(n_jobs=56)]: Done 1346 tasks      | elapsed:   49.3s
[Parallel(n_jobs=56)]: Done 1688 tasks      | elapsed:  1.0min
[Parallel(n_jobs=56)]: Done 2066 tasks      | elapsed:  1.2min
[Parallel(n_jobs=56)]: Done 2480 tasks      | elapsed:  1.4min
[Parallel(n_jobs=56)]: Done 2930 tasks      | elapsed:  1.7min
[Parallel(n_jobs=56)]: Done 3416 tasks      | elapsed:  2.0min
[Parallel(n_jobs=56)]: Done 3938 tasks      | elapsed:  2.3min
[Parallel(n_jobs=56)]: Done 4496 tasks      | elapsed:  2.6min
[Parallel(n_jobs=56)]: Done 5090 tasks      | elapsed:  2.9min
[Parallel(n_jobs=56)]: Done 5720 tasks      | elapsed:  3.3min
[Parallel(n_jobs=56)]: Done 6386 tasks      | elapsed:  3.6min
[Parallel(n_jobs=56)]: Done 7088 tasks      | elapsed:  4.0min
[Parallel(n_jobs=56)]: Done 7826 tasks      | elapsed:  4.4min
[Parallel(n_jobs=56)]: Done 8600 tasks      | elapsed:  4.9min
[Parallel(n_jobs=56)]: Done 9410 tasks      | elapsed:  5.3min
[Parallel(n_jobs=56)]: Done 10000 out of 10000 | elapsed:  5.7min finished
```

In [65]:

```
def dec_CN_boot_wrap(i):
    np.random.seed()
    fake_index = np.random.choice(len(NT_bootstrap_params[0,:]))
    # assume that we have the same number of bootstrap params for each spline 
    fake_NT_params = NT_bootstrap_params[:,fake_index]
    fake_NCHI_params = NCHI_bootstrap_params[:,fake_index]
    
    (dec_CN_params_boot,
     dec_CN_curves_boot,
     dec_CN_residuals_boot) = do_model_analysis('dec', fake_NT_params,
                                                fake_NCHI_params, '4Tcm', 
                                                logT_res = dec_CN_residuals_etc[1],
                                                logitCHI_res = dec_CN_residuals_etc[3],
                                                params_guess = dec_CN_params_hat[2:],
                                                verbose=False)
    
    return (dec_CN_params_boot, dec_CN_curves_boot)

results = Parallel(n_jobs=num_cores, verbose=5)(delayed(dec_CN_boot_wrap)(i) for i in inputs)
dec_CN_bootstrap_params, dec_CN_bootstrap_curves = zip(*results)
# for historical reasons we have to do transposing and axes swapping so these
# object are the shape expected downstream
dec_CN_bootstrap_params = np.array(dec_CN_bootstrap_params).T
dec_CN_bootstrap_curves = np.swapaxes(np.array(dec_CN_bootstrap_curves), 0, 2)
```

```
[Parallel(n_jobs=56)]: Done  50 tasks      | elapsed:    2.4s
[Parallel(n_jobs=56)]: Done 176 tasks      | elapsed:    7.0s
[Parallel(n_jobs=56)]: Done 338 tasks      | elapsed:   13.3s
[Parallel(n_jobs=56)]: Done 536 tasks      | elapsed:   20.2s
[Parallel(n_jobs=56)]: Done 770 tasks      | elapsed:   28.3s
[Parallel(n_jobs=56)]: Done 1040 tasks      | elapsed:   37.8s
[Parallel(n_jobs=56)]: Done 1346 tasks      | elapsed:   48.2s
[Parallel(n_jobs=56)]: Done 1688 tasks      | elapsed:   59.3s
[Parallel(n_jobs=56)]: Done 2066 tasks      | elapsed:  1.2min
[Parallel(n_jobs=56)]: Done 2480 tasks      | elapsed:  1.4min
[Parallel(n_jobs=56)]: Done 2930 tasks      | elapsed:  1.7min
[Parallel(n_jobs=56)]: Done 3416 tasks      | elapsed:  2.0min
[Parallel(n_jobs=56)]: Done 3938 tasks      | elapsed:  2.3min
[Parallel(n_jobs=56)]: Done 4496 tasks      | elapsed:  2.6min
[Parallel(n_jobs=56)]: Done 5090 tasks      | elapsed:  3.0min
[Parallel(n_jobs=56)]: Done 5720 tasks      | elapsed:  3.3min
[Parallel(n_jobs=56)]: Done 6386 tasks      | elapsed:  3.7min
[Parallel(n_jobs=56)]: Done 7088 tasks      | elapsed:  4.1min
[Parallel(n_jobs=56)]: Done 7826 tasks      | elapsed:  4.5min
[Parallel(n_jobs=56)]: Done 8600 tasks      | elapsed:  5.0min
[Parallel(n_jobs=56)]: Done 9410 tasks      | elapsed:  5.5min
[Parallel(n_jobs=56)]: Done 10000 out of 10000 | elapsed:  5.9min finished
```

In [66]:

```
def print_lower_fit_upper_for_model_params(model = 'inc', target = 'E', source = 'N'):
    # model is one of 'inc' or 'dec'
    # target is one of 'E' or 'C',
    # source is one of 'N' or 'C'.
    spec = ''.join([model,'_',target,source])
    bootstrap_params = eval(spec+'_bootstrap_params')
    num_bootstraps = len(bootstrap_params[0,:])
    sorted_straps = np.zeros((5, num_bootstraps))
    for ii in range(5): sorted_straps[ii,:] = np.sort(bootstrap_params[ii,:])
    lowerBounds = sorted_straps[:, np.int(np.ceil(0.025 * num_bootstraps))]
    upperBounds = sorted_straps[:, np.int(np.floor(0.975 * num_bootstraps))]
    dfParam = pd.DataFrame()
    dfParam['model'] = [spec] * 5 # since there are 5 parameters
    if model == 'inc':
        dfParam['params'] = ['T_0', 'CHI_0', 'gamma', 'lambda', 'I_0']
    elif model == 'dec':
        dfParam['params'] = ['T_0', 'CHI_0', 'gamma', 'phi', 'lambda']
    dfParam['lower'] = lowerBounds
    dfParam['fit'] = eval(spec+'_params_hat')
    dfParam['upper'] = upperBounds
    print('for model '+model+', with target population '+target+
          ', and with source population ' +source+',\nthe parameter estimates are:\n')
    print(dfParam)
    print('\nThis model has AIC: '+str(eval(spec+'_residuals_etc')[-1]))
    return dfParam
```

In [67]:

```
inc_EC_param_df = print_lower_fit_upper_for_model_params('inc','E','C')
```

```
for model inc, with target population E, and with source population C,
the parameter estimates are:

    model  params         lower           fit         upper
0  inc_EC     T_0  3.179756e+06  4.250903e+06  5.903536e+06
1  inc_EC   CHI_0  1.885731e-02  2.722944e-02  4.000816e-02
2  inc_EC   gamma  1.342947e-01  2.029390e-01  4.297276e-01
3  inc_EC  lambda  5.871022e-03  1.154274e-02  3.750365e-02
4  inc_EC     I_0  3.326960e+01  6.169453e+05  2.470081e+06

This model has AIC: 124.361135838
```

In [68]:

```
dec_EC_param_df = print_lower_fit_upper_for_model_params('dec','E','C')
```

```
for model dec, with target population E, and with source population C,
the parameter estimates are:

    model  params         lower           fit         upper
0  dec_EC     T_0  3.257658e+06  4.424273e+06  6.092549e+06
1  dec_EC   CHI_0  1.837352e-02  2.747535e-02  4.025201e-02
2  dec_EC   gamma  9.546251e-02  1.724221e-01  3.557725e-01
3  dec_EC     phi -4.168204e-03 -8.222068e-04  4.984964e-03
4  dec_EC  lambda  4.169491e-03  1.088852e-02  2.438871e-02

This model has AIC: 124.434234884
```

In [69]:

```
inc_EN_param_df = print_lower_fit_upper_for_model_params('inc','E','N')
```

```
for model inc, with target population E, and with source population N,
the parameter estimates are:

    model  params         lower           fit         upper
0  inc_EN     T_0  4.581986e+06  5.515871e+06  6.815907e+06
1  inc_EN   CHI_0  1.847175e-02  2.570547e-02  3.506496e-02
2  inc_EN   gamma  1.161277e-03  1.724671e-03  2.477512e-03
3  inc_EN  lambda -1.905994e-03  1.508945e-03  5.357408e-03
4  inc_EN     I_0  4.569151e+03  5.333832e+06  6.063871e+06

This model has AIC: 113.255052454
```

In [70]:

```
dec_EN_param_df = print_lower_fit_upper_for_model_params('dec','E','N')
```

```
for model dec, with target population E, and with source population N,
the parameter estimates are:

    model  params         lower           fit         upper
0  dec_EN     T_0  4.519033e+06  5.550482e+06  6.855384e+06
1  dec_EN   CHI_0  1.814721e-02  2.558272e-02  3.461940e-02
2  dec_EN   gamma  9.203824e-04  2.200561e-03  5.828575e-03
3  dec_EN     phi -2.570637e-03  2.162332e-03  8.200303e-03
4  dec_EN  lambda -1.290795e-03  2.232532e-04  1.786218e-03

This model has AIC: 113.09529852
```

In [71]:

```
inc_CN_param_df = print_lower_fit_upper_for_model_params('inc','C','N')
```

```
for model inc, with target population C, and with source population N,
the parameter estimates are:

    model  params          lower            fit         upper
0  inc_CN     T_0  691223.032230  926743.512941  1.314958e+06
1  inc_CN   CHI_0       0.051271       0.074153  1.091461e-01
2  inc_CN   gamma       0.000279       0.000452  8.517734e-04
3  inc_CN  lambda       0.011422       0.019061  3.787519e-02
4  inc_CN     I_0   74215.110437  145532.925504  2.213930e+05

This model has AIC: 145.147946561
```

In [72]:

```
dec_CN_param_df = print_lower_fit_upper_for_model_params('dec','C','N')
```

```
for model dec, with target population C, and with source population N,
the parameter estimates are:

    model  params          lower            fit          upper
0  dec_CN     T_0  502603.096668  689128.594213  932976.112754
1  dec_CN   CHI_0       0.052733       0.072587       0.107079
2  dec_CN   gamma       0.000471       0.001319       0.004157
3  dec_CN     phi       0.003706       0.009701       0.016771
4  dec_CN  lambda       0.005110       0.007149       0.009647

This model has AIC: 153.425705537
```

In [73]:

```
param_df = pd.concat([inc_EC_param_df, dec_EC_param_df, 
                      inc_EN_param_df, dec_EN_param_df,
                      inc_CN_param_df, dec_CN_param_df])
print(param_df)
```

```
    model  params         lower           fit         upper
0  inc_EC     T_0  3.179756e+06  4.250903e+06  5.903536e+06
1  inc_EC   CHI_0  1.885731e-02  2.722944e-02  4.000816e-02
2  inc_EC   gamma  1.342947e-01  2.029390e-01  4.297276e-01
3  inc_EC  lambda  5.871022e-03  1.154274e-02  3.750365e-02
4  inc_EC     I_0  3.326960e+01  6.169453e+05  2.470081e+06
0  dec_EC     T_0  3.257658e+06  4.424273e+06  6.092549e+06
1  dec_EC   CHI_0  1.837352e-02  2.747535e-02  4.025201e-02
2  dec_EC   gamma  9.546251e-02  1.724221e-01  3.557725e-01
3  dec_EC     phi -4.168204e-03 -8.222068e-04  4.984964e-03
4  dec_EC  lambda  4.169491e-03  1.088852e-02  2.438871e-02
0  inc_EN     T_0  4.581986e+06  5.515871e+06  6.815907e+06
1  inc_EN   CHI_0  1.847175e-02  2.570547e-02  3.506496e-02
2  inc_EN   gamma  1.161277e-03  1.724671e-03  2.477512e-03
3  inc_EN  lambda -1.905994e-03  1.508945e-03  5.357408e-03
4  inc_EN     I_0  4.569151e+03  5.333832e+06  6.063871e+06
0  dec_EN     T_0  4.519033e+06  5.550482e+06  6.855384e+06
1  dec_EN   CHI_0  1.814721e-02  2.558272e-02  3.461940e-02
2  dec_EN   gamma  9.203824e-04  2.200561e-03  5.828575e-03
3  dec_EN     phi -2.570637e-03  2.162332e-03  8.200303e-03
4  dec_EN  lambda -1.290795e-03  2.232532e-04  1.786218e-03
0  inc_CN     T_0  6.912230e+05  9.267435e+05  1.314958e+06
1  inc_CN   CHI_0  5.127133e-02  7.415340e-02  1.091461e-01
2  inc_CN   gamma  2.793561e-04  4.522649e-04  8.517734e-04
3  inc_CN  lambda  1.142220e-02  1.906054e-02  3.787519e-02
4  inc_CN     I_0  7.421511e+04  1.455329e+05  2.213930e+05
0  dec_CN     T_0  5.026031e+05  6.891286e+05  9.329761e+05
1  dec_CN   CHI_0  5.273334e-02  7.258743e-02  1.070787e-01
2  dec_CN   gamma  4.706148e-04  1.319469e-03  4.156600e-03
3  dec_CN     phi  3.706479e-03  9.700685e-03  1.677135e-02
4  dec_CN  lambda  5.110403e-03  7.149204e-03  9.647075e-03
```

Lets look at a histogram of those parameter estimate bootstraps:

In [74]:

```
def make_param_histograms(model = 'inc', target = 'E', source = 'N', 
                          title = 'Tem with Nai as source, incumbent model'):
    spec = ''.join([model,'_',target,source])
    bootstrap_params = eval(spec+'_bootstrap_params')
    num_bootstraps = len(bootstrap_params[0,:])
    
    figT_0, axT_0 = plt.subplots()
    axT_0.hist(bootstrap_params[0,:], bins=50)
    axT_0.vlines(eval(spec+'_params_hat')[0],0,0.5*num_bootstraps)
    axT_0.set_xlabel('T_0 estimates')
    axT_0.set_ylabel('counts')
    axT_0.set_title('Histogram of T_0 estimates: ' + title)

    figCHI_0, axCHI_0 = plt.subplots()
    axCHI_0.hist(bootstrap_params[1,:], bins=50)
    axCHI_0.vlines(eval(spec+'_params_hat')[1],0,0.5*num_bootstraps)
    axCHI_0.set_xlabel('CHI_0 estimates')
    axCHI_0.set_ylabel('counts')
    axCHI_0.set_title('Histogram of CHI_0 estimates:' + title)

    figgam, axgam = plt.subplots()
    axgam.hist(bootstrap_params[2,:], bins=50)
    axgam.vlines(eval(spec+'_params_hat')[2],0,0.5*num_bootstraps)
    axgam.set_xlabel('gamma estimates')
    axgam.set_ylabel('counts')
    axgam.set_title('Histogram of gamma estimates:' + title)
    
    if model == 'inc':
        figlam, axlam = plt.subplots()
        axlam.hist(bootstrap_params[3,:], bins=50)
        axlam.vlines(eval(spec+'_params_hat')[3],0,0.5*num_bootstraps)
        axlam.set_xlabel('lambda estimates')
        axlam.set_ylabel('counts')
        axlam.set_title('Histogram of lambda estimates: ' + title)
        
        figI_0, axI_0 = plt.subplots()
        axI_0.hist(bootstrap_params[4,:], bins=50)
        axI_0.vlines(eval(spec+'_params_hat')[4],0,0.5*num_bootstraps)
        axI_0.set_xlabel('resistant estimates')
        axI_0.set_ylabel('counts')
        axI_0.set_title('Histogram of incumbent estimates: ' + title)
        
        figMM, axMM = plt.subplots()
        xy = np.vstack([bootstrap_params[4,:],bootstrap_params[3,:]])
        z = gaussian_kde(xy)(xy)
        axMM.scatter(bootstrap_params[4,:], bootstrap_params[3,:], c=z,
                     s=10, edgecolor='')
        axMM.set_xlabel('resistant estimate')
        axMM.set_ylabel('lambda estimates')
        axMM.set_title('paired resistant and lambda estimates')
    elif model == 'dec':
        figphi, axphi = plt.subplots()
        axphi.hist(bootstrap_params[3,:], bins=50)
        axphi.vlines(eval(spec+'_params_hat')[3],0,0.5*num_bootstraps)
        axphi.set_xlabel('phi estimates')
        axphi.set_ylabel('counts')
        axphi.set_title('Histogram of phi estimates: ' + title)

        figlam, axlam = plt.subplots()
        axlam.hist(bootstrap_params[4,:], bins=50)
        axlam.vlines(eval(spec+'_params_hat')[4],0,0.5*num_bootstraps)
        axlam.set_xlabel('lambda estimates')
        axlam.set_ylabel('counts')
        axlam.set_title('Histogram of lambda estimates: ' + title)
```

In [75]:

```
make_param_histograms('inc','E','N', 'Tem with Nai as source, incumbent model')
```

In [76]:

```
make_param_histograms('dec','E','N', 'Tem with Nai as source, decay model')
```

In [77]:

```
make_param_histograms('inc','E','C', 'Tem with Tcm as source, incumbent model')
```

In [78]:

```
make_param_histograms('dec','E','C', 'Tem with Tcm as source, decay model')
```

In [79]:

```
make_param_histograms('inc','C','N', 'Tcm with Nai as source, incumbent model')
```

In [80]:

```
make_param_histograms('dec','C','N', 'Tcm with Nai as source, decay model')
```

Now we use the bootstraps to see how our uncertainty about the parameters is reflected in our fits to the data. Additionally we are interested in the flow rate into the memory compartments. So we plot instantaneous total inflow as a proportion of the instanteous total memory cells M for both central and effector CD4+ cells. To make this more readily undertandable we also plot the "weekly turnover" i.e. the proportion of the cells that will have arrived within the past week, a week from now.

In [81]:

```
df_curve_bounds = pd.DataFrame()
dpBMT = ddTarget['days.post.bmt']
df_curve_bounds['days.post.bmt'] = np.arange(np.min(dpBMT), np.max(dpBMT)+1)
df_curve_bounds['mouse.age.days'] = df_curve_bounds['days.post.bmt'] + 56
```

In [82]:

```
def make_figs_of_bounds(model = 'inc', target = 'E', source = 'N',
                        title = 'Effector Memory with Naive as source, incumbent model',
                        plotting_data = df_curve_bounds):
    T = ddTarget['TOTAL.4T'+target.lower()+'m']
    CHI = ddTarget['NCHI.4T'+target.lower()+'m']
    spec = ''.join([model,'_',target,source])
    (T_hat_smooth, 
     CHI_hat_smooth, 
     In_smooth, 
     Turn_smooth,
     Turn_no_inc_smooth,
     Inc_prop_smooth) = eval(spec+'_curves')
    dpBMT = ddTarget['days.post.bmt']
    ageBMT = 56
    
    bootstrap_curves = eval(spec+'_bootstrap_curves') #[time x curve x bootstrap]
    smoothdpBMT = np.arange(np.min(dpBMT), np.max(dpBMT)+1)
    if not len(smoothdpBMT) == bootstrap_curves.shape[0]:
        print('curves aren\'t the right length')
    num_bootstraps = bootstrap_curves.shape[-1] 
    bound_indices = [np.int(np.floor(0.975 * num_bootstraps)), np.int(np.ceil(0.025 * num_bootstraps))]

    T_hat_bounds           = np.zeros((2, len(smoothdpBMT)))
    CHI_hat_bounds         = np.zeros((2, len(smoothdpBMT)))
    In_hat_bounds          = np.zeros((2, len(smoothdpBMT)))
    Turn_hat_bounds        = np.zeros((2, len(smoothdpBMT)))
    Turn_no_inc_hat_bounds = np.zeros((2, len(smoothdpBMT)))
    Inc_prop_hat_bounds    = np.zeros((2, len(smoothdpBMT)))
        
    for ii, tt in enumerate(smoothdpBMT):
        sorted_T_tt = np.sort(bootstrap_curves[ii, 0, :])
        sorted_CHI_tt = np.sort(bootstrap_curves[ii, 1, :])
        sorted_In_tt = np.sort(bootstrap_curves[ii, 2, :])
        sorted_Turn_tt = np.sort(bootstrap_curves[ii, 3, :])
        sorted_Turn_no_inc_tt = np.sort(bootstrap_curves[ii, 4, :])
        sorted_Inc_prop_tt = np.sort(bootstrap_curves[ii, 5, :])
    
        T_hat_bounds[:,ii] = sorted_T_tt[bound_indices]
        CHI_hat_bounds[:,ii] = sorted_CHI_tt[bound_indices]
        In_hat_bounds[:,ii] = sorted_In_tt[bound_indices]
        Turn_hat_bounds[:,ii] = sorted_Turn_tt[bound_indices]
        Turn_no_inc_hat_bounds[:,ii] = sorted_Turn_no_inc_tt[bound_indices]
        Inc_prop_hat_bounds[:,ii] = sorted_Inc_prop_tt[bound_indices]
            
    figT, axT = plt.subplots()
    axT.fill_between(smoothdpBMT, T_hat_bounds[1,:], T_hat_bounds[0,:],
                     facecolor=sns.color_palette()[4], interpolate=True)
    axT.scatter(dpBMT, T)
    axT.plot(smoothdpBMT, T_hat_smooth)
    axT.set_xlabel('days post bone marrow transplant')
    axT.set_ylabel('cell counts')
    axT2 = axT.twiny()
    axT2.set_xlim(axT.get_xlim())
    axT2.set_xticklabels((axT.get_xticks() + ageBMT).astype(int))
    axT2.set_xlabel('mouse age in days')
    axT2.set_title('Counts: ' + title, y=1.1)
    
    figCHI, axCHI = plt.subplots()
    axCHI.fill_between(smoothdpBMT, CHI_hat_bounds[1,:], CHI_hat_bounds[0,:], 
                       facecolor=sns.color_palette()[4], interpolate=True)
    axCHI.scatter(dpBMT, CHI)
    axCHI.plot(smoothdpBMT, CHI_hat_smooth)
    axCHI.set_xlabel('days post bone marrow transplant')
    axCHI.set_ylabel('Normalized Chimerism')
    axCHI2 = axCHI.twiny()
    axCHI2.set_xlim(axCHI.get_xlim())
    axCHI2.set_xticklabels((axCHI.get_xticks() + ageBMT).astype(int))
    axCHI2.set_xlabel('mouse age in days')
    axCHI2.set_title('Chimerism: '+title, y=1.1)
    
    figIn, axIn = plt.subplots()
    axIn.fill_between(smoothdpBMT, In_hat_bounds[1,:], In_hat_bounds[0,:],
                      facecolor=sns.color_palette()[4], interpolate=True)
    axIn.plot(smoothdpBMT, In_smooth, label='estimate')
    axIn.set_xlabel('days post bone marrow transplant')
    axIn.set_ylabel('proportional inflow')
    axIn2 = axIn.twiny()
    axIn2.set_xlim(axIn.get_xlim())
    axIn2.set_xticklabels((axIn.get_xticks() + ageBMT).astype(int))
    axIn2.set_xlabel('mouse age in days')
    axIn2.set_title('Proportional inflow: '+title, y=1.1)

    figTurn, axTurn = plt.subplots()
    axTurn.fill_between(smoothdpBMT, Turn_hat_bounds[1,:], Turn_hat_bounds[0,:], 
                        facecolor=sns.color_palette()[4], interpolate=True)
    axTurn.plot(smoothdpBMT, Turn_smooth, label='estimate')
    axTurn.set_xlabel('days post bone marrow transplant')
    axTurn.set_ylabel('weekly turnover fraction')
    axTurn2 = axTurn.twiny()
    axTurn2.set_xlim(axTurn.get_xlim())
    axTurn2.set_xticklabels((axTurn.get_xticks() + ageBMT).astype(int))
    axTurn2.set_xlabel('mouse age in days')
    axTurn2.set_title('Weekly turnover:'+title, y=1.1)
    
    if model == 'inc':
        figTurn_no_inc, axTurn_no_inc = plt.subplots()
        axTurn_no_inc.fill_between(smoothdpBMT, Turn_no_inc_hat_bounds[1,:], 
                                   Turn_no_inc_hat_bounds[0,:], 
                                   facecolor=sns.color_palette()[4], interpolate=True)
        axTurn_no_inc.plot(smoothdpBMT, Turn_no_inc_smooth, label='estimate')
        axTurn_no_inc.set_xlabel('days post bone marrow transplant')
        axTurn_no_inc.set_ylabel('weekly turnover fraction of replaceable')
        axTurn_no_inc2 = axTurn_no_inc.twiny()
        axTurn_no_inc2.set_xlim(axTurn_no_inc.get_xlim())
        axTurn_no_inc2.set_xticklabels((axTurn_no_inc.get_xticks() + ageBMT).astype(int))
        axTurn_no_inc2.set_xlabel('mouse age in days')
        axTurn_no_inc2.set_title('Weekly turnover of replaceable:'+title, y=1.1)
        
        figInc_prop, axInc_prop = plt.subplots()
        axInc_prop.fill_between(smoothdpBMT, Inc_prop_hat_bounds[1,:], 
                                   Inc_prop_hat_bounds[0,:], 
                                   facecolor=sns.color_palette()[4], interpolate=True)
        axInc_prop.plot(smoothdpBMT, Inc_prop_smooth, label='estimate')
        axInc_prop.set_xlabel('days post bone marrow transplant')
        axInc_prop.set_ylabel('Proportion resistant cells')
        axInc_prop2 = axInc_prop.twiny()
        axInc_prop2.set_xlim(axInc_prop.get_xlim())
        axInc_prop2.set_xticklabels((axInc_prop.get_xticks() + ageBMT).astype(int))
        axInc_prop2.set_xlabel('mouse age in days')
        axInc_prop2.set_title('Proportion of resistant cells:'+title, y=1.1)
    
    #put these curves in a dataframe that we can export to csv for plotting
    plotting_data.loc[:,spec+'_counts_upp']   = T_hat_bounds[0,:]
    plotting_data.loc[:,spec+'_counts_fit']   = T_hat_smooth
    plotting_data.loc[:,spec+'_counts_low']   = T_hat_bounds[1,:]
    
    plotting_data.loc[:,spec+'_chi_upp']      = CHI_hat_bounds[0,:]
    plotting_data.loc[:,spec+'_chi_fit']      = CHI_hat_smooth
    plotting_data.loc[:,spec+'_chi_low']      = CHI_hat_bounds[1,:]
    
    plotting_data.loc[:,spec+'_inflow_upp']   = In_hat_bounds[0,:]
    plotting_data.loc[:,spec+'_inflow_fit']   = In_smooth
    plotting_data.loc[:,spec+'_inflow_low']   = In_hat_bounds[1,:]
    
    plotting_data.loc[:,spec+'_turnover_upp'] = Turn_hat_bounds[0,:]
    plotting_data.loc[:,spec+'_turnover_fit'] = Turn_smooth
    plotting_data.loc[:,spec+'_turnover_low'] = Turn_hat_bounds[1,:]
    
    plotting_data.loc[:,spec+'_turnover_displaceable_upp'] = Turn_no_inc_hat_bounds[0,:]
    plotting_data.loc[:,spec+'_turnover_displaceable_fit'] = Turn_no_inc_smooth
    plotting_data.loc[:,spec+'_turnover_displaceable_low'] = Turn_no_inc_hat_bounds[1,:]
    
    plotting_data.loc[:,spec+'_incumbent_prop_upp'] = Inc_prop_hat_bounds[0,:]
    plotting_data.loc[:,spec+'_incumbent_prop_fit'] = Inc_prop_smooth
    plotting_data.loc[:,spec+'_incumbent_prop_low'] = Inc_prop_hat_bounds[1,:]
```

In [83]:

```
make_figs_of_bounds(model = 'dec',
                    target='E',
                    source='N',
                    title='Effector Memory with Niave as source and declining recruitment',
                    plotting_data = df_curve_bounds)
```

In [84]:

```
make_figs_of_bounds(model = 'inc',
                    target='E',
                    source='N',
                    title='Effector Memory with Naive as source and resistant memory',
                    plotting_data = df_curve_bounds)
```

In [85]:

```
make_figs_of_bounds(model = 'dec',
                    target='E',
                    source='C',
                    title='Effector Memory with Central Memory as source and declining recruitment',
                    plotting_data = df_curve_bounds)
```

In [86]:

```
make_figs_of_bounds(model = 'inc',
                    target='E',
                    source='C',
                    title='Effector Memory with Central Memory as source and resistant memory',
                    plotting_data = df_curve_bounds)
```

In [87]:

```
make_figs_of_bounds(model = 'dec',
                    target='C',
                    source='N',
                    title='Effector Memory with Niave as source and declining recruitment',
                    plotting_data = df_curve_bounds)
```

In [88]:

```
make_figs_of_bounds(model = 'inc',
                    target='C',
                    source='N',
                    title='Central Memory with Niave as source and resistant memory',
                    plotting_data = df_curve_bounds)
```

In [89]:

```
# If so inclined, export this data frame as csv for plotting
#df_curve_bounds.to_csv('plotsForSixModels.csv')
```

In [90]:

```
def get_prop_inflow_est_at_time(model = 'inc', target = 'E', source = 'N', days_post_bmt = 42):
    # want 98 day old mice
    # 98 - 56 = 42 days post BMT
    spec = ''.join([model,'_',target,source])
    In_smooth = eval(spec+'_curves')[2]
    Turn_smooth = eval(spec+'_curves')[3]
    dpBMT = ddTarget['days.post.bmt']
    ageBMT = 56
    
    bootstrap_curves = eval(spec+'_bootstrap_curves') #[time x curve x bootstrap]
    smoothdpBMT = np.arange(np.min(dpBMT), np.max(dpBMT)+1)
    if not len(smoothdpBMT) == bootstrap_curves.shape[0]:
        print('curves aren\'t the right length')
    num_bootstraps = bootstrap_curves.shape[-1] 
    
    In_hat_bounds   = np.zeros((2, len(smoothdpBMT)))
    Turn_hat_bounds = np.zeros((2, len(smoothdpBMT)))
    bound_indices = [np.int(np.floor(0.975 * num_bootstraps)), np.int(np.ceil(0.025 * num_bootstraps))]
    
    for ii, tt in enumerate(smoothdpBMT):
        sorted_In_tt = np.sort(bootstrap_curves[ii, 2, :])
        sorted_Turn_tt = np.sort(bootstrap_curves[ii, 3, :])
        In_hat_bounds[:,ii] = sorted_In_tt[bound_indices]
        Turn_hat_bounds[:,ii] = sorted_Turn_tt[bound_indices]
        if tt == days_post_bmt:
            print('95% Bootstrap Interval of proportional inflow at ' + 
                  str(days_post_bmt)+ ' days post bone marrow transplant is:' + str(In_hat_bounds[:,ii][::-1]))
            print('Best fit is: '+str(In_smooth[ii]))
            
            print('95% Bootstrap Interval for weekly turnover at ' + 
                  str(days_post_bmt)+ ' days post bone marrow transplant is:' + str(Turn_hat_bounds[:,ii][::-1]))
            print('Best fit is: '+str(Turn_smooth[ii]))
            
    figIn, axIn = plt.subplots()
    axIn.fill_between(smoothdpBMT, In_hat_bounds[1,:], In_hat_bounds[0,:],
                      facecolor=sns.color_palette()[4], interpolate=True)
    axIn.plot(smoothdpBMT, In_smooth, label='estimate')
    axIn.vlines(days_post_bmt,0,0.05)
    axIn.set_xlabel('days post bone marrow transplant')
    axIn.set_ylabel('proportional inflow')
    axIn2 = axIn.twiny()
    axIn2.set_xlim(axIn.get_xlim())
    axIn2.set_xticklabels((axIn.get_xticks() + ageBMT).astype(int))
    axIn2.set_xlabel('mouse age in days')
    axIn2.set_title('Proportional inflow: '+str(spec), y=1.1)
```

In [91]:

```
get_prop_inflow_est_at_time(model = 'inc', target = 'E', source = 'N', days_post_bmt = 42)
```

```
95% Bootstrap Interval of proportional inflow at 42 days post bone marrow transplant is:[ 0.00652455  0.01377898]
Best fit is: 0.00970753337618
95% Bootstrap Interval for weekly turnover at 42 days post bone marrow transplant is:[ 0.04302879  0.08560777]
Best fit is: 0.0622555295278
```

In [92]:

```
get_prop_inflow_est_at_time(model = 'dec', target = 'E', source = 'N', days_post_bmt = 42)
```

```
95% Bootstrap Interval of proportional inflow at 42 days post bone marrow transplant is:[ 0.00627994  0.01582503]
Best fit is: 0.00995838167906
95% Bootstrap Interval for weekly turnover at 42 days post bone marrow transplant is:[ 0.04168152  0.09610928]
Best fit is: 0.0636557762778
```

In [93]:

```
get_prop_inflow_est_at_time(model = 'inc', target = 'E', source = 'C', days_post_bmt = 42)
```

```
95% Bootstrap Interval of proportional inflow at 42 days post bone marrow transplant is:[ 0.02575306  0.08196383]
Best fit is: 0.0411324486941
95% Bootstrap Interval for weekly turnover at 42 days post bone marrow transplant is:[ 0.15488611  0.37026643]
Best fit is: 0.227120803027
```

In [94]:

```
get_prop_inflow_est_at_time(model = 'dec', target = 'E', source = 'C', days_post_bmt = 42)
```

```
95% Bootstrap Interval of proportional inflow at 42 days post bone marrow transplant is:[ 0.02130834  0.06116685]
Best fit is: 0.0363952937879
95% Bootstrap Interval for weekly turnover at 42 days post bone marrow transplant is:[ 0.13325371  0.30970462]
Best fit is: 0.208454791931
```

In [95]:

```
get_prop_inflow_est_at_time(model = 'inc', target = 'C', source = 'N', days_post_bmt = 42)
```

```
95% Bootstrap Interval of proportional inflow at 42 days post bone marrow transplant is:[ 0.00931567  0.02702874]
Best fit is: 0.0151513091494
95% Bootstrap Interval for weekly turnover at 42 days post bone marrow transplant is:[ 0.0625666   0.16475863]
Best fit is: 0.0982160449462
```

In [96]:

```
get_prop_inflow_est_at_time(model = 'dec', target = 'C', source = 'N', days_post_bmt = 42)
```

```
95% Bootstrap Interval of proportional inflow at 42 days post bone marrow transplant is:[ 0.0129109   0.04256151]
Best fit is: 0.0229745080905
95% Bootstrap Interval for weekly turnover at 42 days post bone marrow transplant is:[ 0.08221365  0.2216546 ]
Best fit is: 0.135328199047
```

As a quick sanity check we also make a point estimate of initial inflow into memory simply using the empirical slope of the Chimerism curve at the first two time observations. We find that this point estimate is in the same range as that found using our mechanistic ODE models.

In [97]:

```
def fit_dCHI_dt(CHI_0_obs, CHI_1_obs):
    #fit effector memory with naive as source
    CHI_0_hat = np.mean(CHI_0_obs)
    CHI_1_hat = np.mean(CHI_1_obs)
    params = (CHI_0_hat, CHI_1_hat)
    residuals = np.hstack((CHI_0_obs - CHI_0_hat, CHI_1_obs - CHI_1_hat))
    cost = np.sum(residuals**2)
    return params, residuals, cost
```

In [98]:

```
def do_simple_analysis(SourceCHI_params = NCHI_params_hat,
                       target = '4Tem',
                       title=''):
    if not target in ['4Tem', '4Tcm']:
        print('invalid choice of target should be 4Tem or 4Tcm')
    dpBMT = ddTarget['days.post.bmt']
    dpBMT_0, dpBMT_1 = np.sort(np.unique(dpBMT))[[0,1]]
    init_dpBMT_indices = np.array(dpBMT == dpBMT_0)
    second_dpBMT_indices = np.array(dpBMT == dpBMT_1)
    CHI = ddTarget['NCHI.'+target]
    CHI_0 = ddTarget['NCHI.'+target].values[init_dpBMT_indices]
    CHI_1 = ddTarget['NCHI.'+target].values[second_dpBMT_indices]
    ageBMT = ddTarget['age.at.bmt'].values[0]
    # age at BMT should be 56 for all of these mice

    params_hat, residuals, cost1 = fit_dCHI_dt(CHI_0, CHI_1)
    CHI_0_hat, CHI_1_hat = params_hat
    SourceCHI_0 = logistic_model(SourceCHI_params, dpBMT_0)
    In_t0_hat = (CHI_1_hat - CHI_0_hat) / (dpBMT_1 - dpBMT_0) / (SourceCHI_0 - CHI_0_hat)
    
    fig, ax = plt.subplots()
    ax.scatter([dpBMT_0]*len(CHI_0), CHI_0)
    ax.scatter([dpBMT_1]*len(CHI_1), CHI_1)
    ax.plot([dpBMT_0, dpBMT_1],[CHI_0_hat, CHI_1_hat])
    ax.set_xlabel('days post bone marrow transplant')
    ax.set_ylabel('Chimerism') 
    ax2 = ax.twiny()
    ax2.set_xlim(ax.get_xlim())
    ax2.set_xticklabels((ax.get_xticks() + ageBMT).astype(int))
    ax2.set_xlabel('mouse age in days')
    ax2.set_title('Proportional inflow: '+title, y=1.1) 
    
    print('Proportional Inflow at t_0 point estimate of: '+str(In_t0_hat))
```

In [99]:

```
do_simple_analysis(SourceCHI_params = NCHI_params_hat, target='4Tem', 
                   title='Effector Memory with Naive as Source')
```

```
Proportional Inflow at t_0 point estimate of: 0.012296867934
```

In [100]:

```
do_simple_analysis(SourceCHI_params = NCHI_params_hat, target='4Tcm',
                   title='Central Memory with Naive as source')
```

```
Proportional Inflow at t_0 point estimate of: 0.0249181756171
```

In [101]:

```
do_simple_analysis(SourceCHI_params = CCHI_spline_params_hat, target='4Tem',
                   title='Effector Memory with Central Memory as source')
```

```
Proportional Inflow at t_0 point estimate of: 0.0745491224185
```
