# Supplementary figures and images for: Memory CD4 T cell subsets are kinetically heterogeneous and replenished from naive T cells at high levels
Source: eLife. 2017 Mar 10;6:e23013. doi: 10.7554/eLife.23013 (PMC5426903; doi:10.7554/eLife.23013)

4cm brdu+%

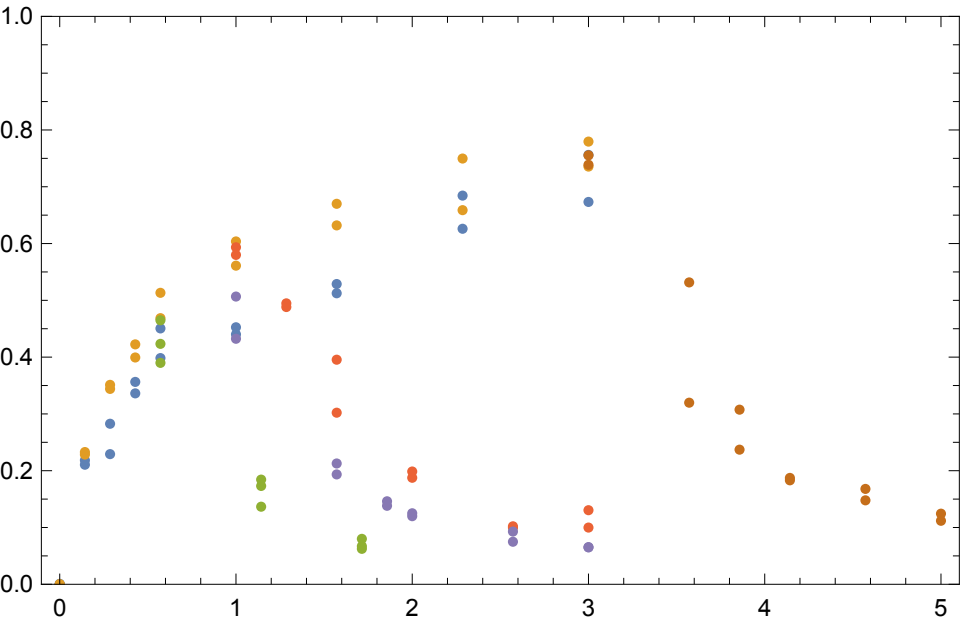

Supplement: Figure 4—source data 1. — DOI: http://dx.doi.org/10.7554/eLife.23013.014 [file elife-23013-fig4-data1.zip › 4Tcm Brdu+.pdf]

4cm Ki67Pos

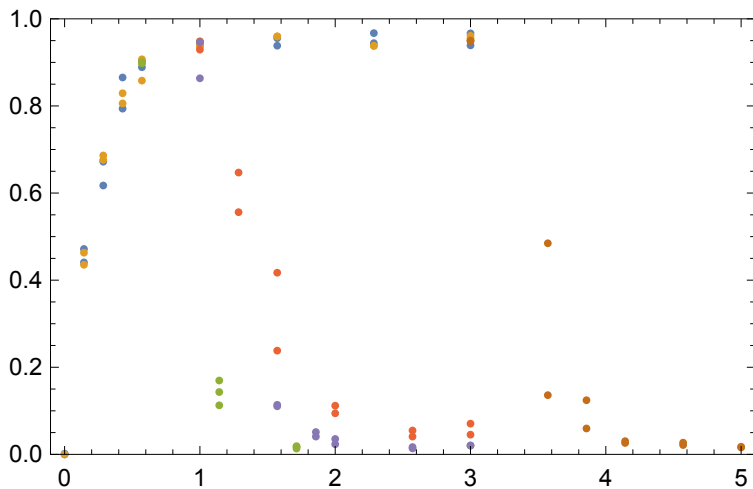

4cm Ki67Neg

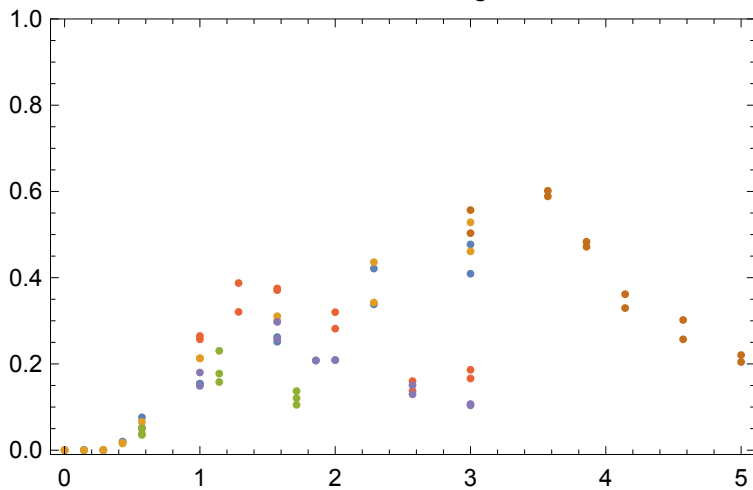

Supplement: Figure 4—source data 1. — DOI: http://dx.doi.org/10.7554/eLife.23013.014 [file elife-23013-fig4-data1.zip › 4TcmDataImg.pdf]

4em brdu+%

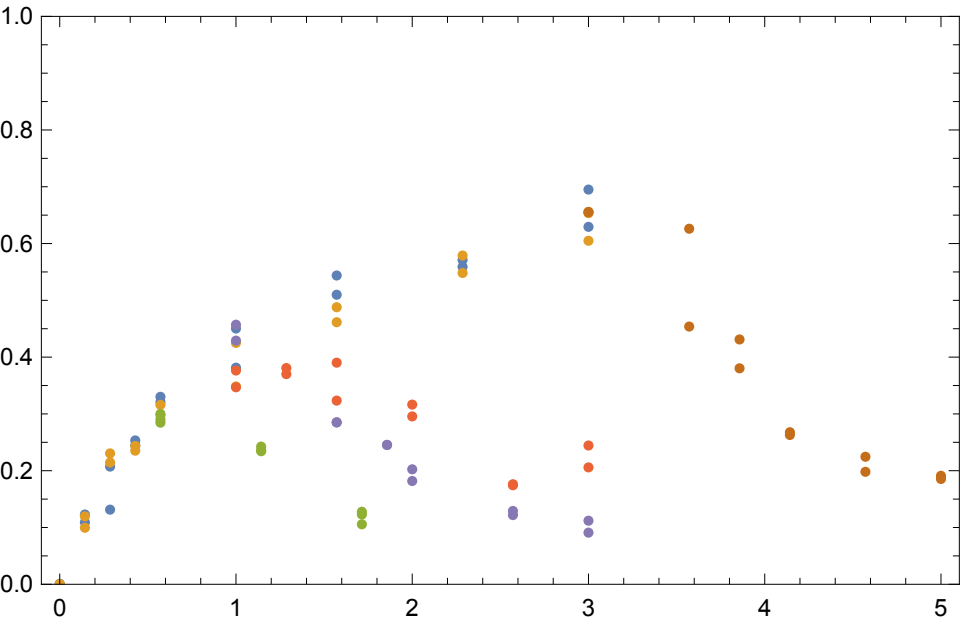

Supplement: Figure 4—source data 1. — DOI: http://dx.doi.org/10.7554/eLife.23013.014 [file elife-23013-fig4-data1.zip › 4Tem Brdu+.pdf]

4em Ki67Pos

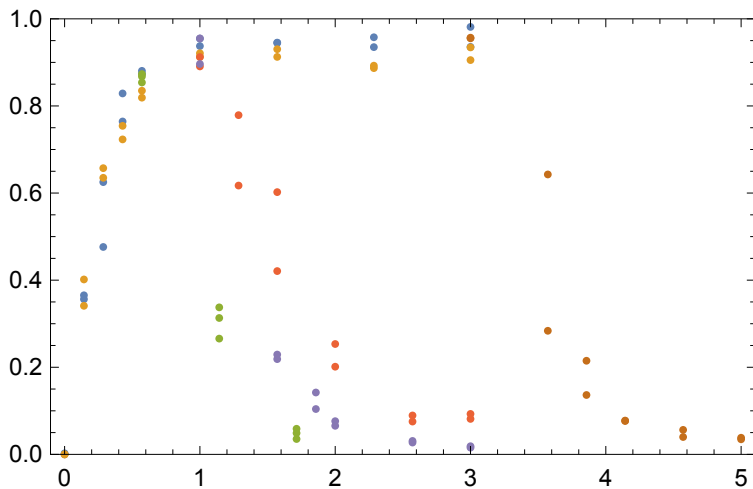

4em Ki67Neg

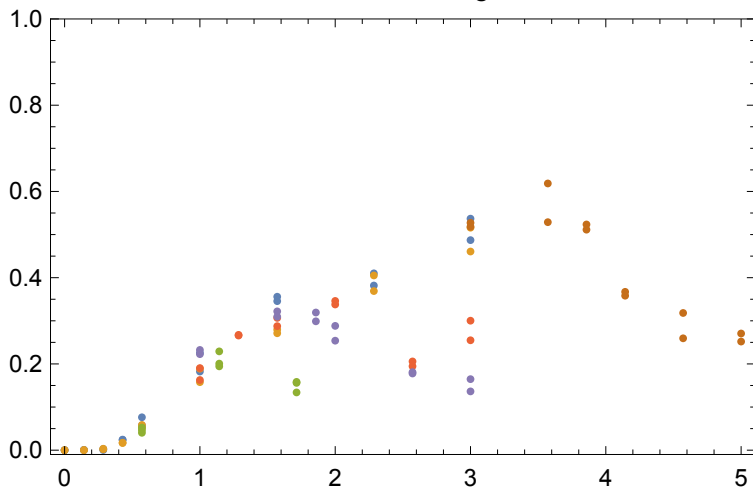

Supplement: Figure 4—source data 1. — DOI: http://dx.doi.org/10.7554/eLife.23013.014 [file elife-23013-fig4-data1.zip › 4TemDataImg.pdf]
