# Supplementary material for: Memory CD4 T cell subsets are kinetically heterogeneous and replenished from naive T cells at high levels
Source: eLife. 2017 Mar 10;6:e23013. doi: 10.7554/eLife.23013 (PMC5426903; doi:10.7554/eLife.23013)
Supplement: Figure 4—source data 2. — DOI: http://dx.doi.org/10.7554/eLife.23013.015 [file elife-23013-fig4-data2.zip › BrdU-Ki67-analysis-code/Data/8TcmDataImg.pdf]

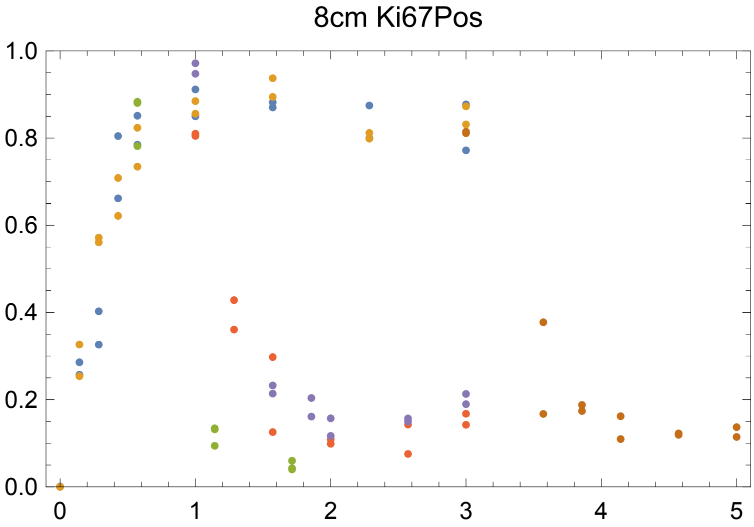
8cm Ki67Pos
1.0
0.8
0.6
0.4
0.2
0.0
0
1
2
3
4
5

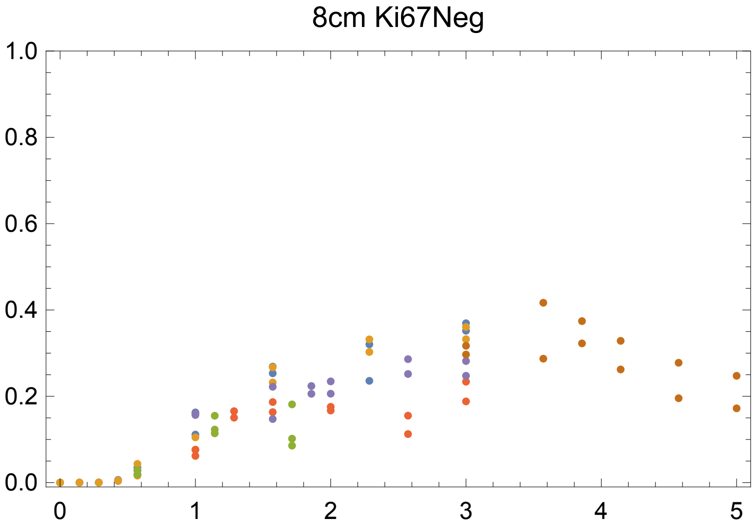
8cm Ki67Neg
1.0
0.8
0.6
0.4
0.2
0.0
0
1
2
3
4
5
